# Development of the Local Food Systems Policy Index (Local Food-EPI+) tool and assessment process to benchmark the implementation of local government policies for creating healthy, equitable and environmentally sustainable food systems

**DOI:** 10.1017/S136898002400140X

**Published:** 2024-10-02

**Authors:** Oriana Ruffini, Chanel Relf, Davina Mann, Miranda R Blake, Amy Carrad, Belinda Reeve, Liza Barbour, Lana Vanderlee, Stefanie Vandevijvere, Gary Sacks

**Affiliations:** 1 Global Centre for Preventive Health and Nutrition, Institute for Health Transformation, Deakin University, Geelong, VIC, Australia; 2 City of Greater Bendigo, Bendigo, VIC, Australia; 3 National Health and Medical Research Council, Canberra, ACT, Australia; 4 Australian Research Centre for Health Equity, Australian National University, Canberra, ACT, Australia; 5 The University of Sydney Law School, University of Sydney, Sydney, NSW, Australia; 6 Department of Nutrition, Dietetics & Food, Monash University, Clayton, VIC, Australia; 7 School of Nutrition, Université Laval, Quebec City, Quebec, Canada; 8 Department of Epidemiology and Public Health, Sciensano, Brussels, Belgium

**Keywords:** Food systems, Food environments, Environmental sustainability, Equity, Policy, Benchmarking, Assessment, Tool, Local government, Council, Planetary health, Australia

## Abstract

**Objective:**

Local governments have an important role to play in creating healthy, equitable and environmentally sustainable food systems. This study aimed to develop and pilot a tool and process for local governments in Australia to benchmark their policies for creating healthy, equitable and environmentally sustainable food systems.

**Design:**

The Healthy Food Environment Policy Index (Food-EPI), developed in 2013 for national governments, was tailored to develop the Local Food Systems Policy Index (Local Food-EPI+) tool for local governments. To incorporate environmental sustainability and the local government context, this process involved a literature review and collaboration with an international and domestic expert advisory committee (*n* 35) and local government officials.

**Setting:**

Local governments.

**Results:**

The tool consists of sixty-one indicators across ten food policy domains (weighted based on relative importance): leadership; governance; funding and resources; monitoring and intelligence; food production and supply chain; food promotion; food provision and retail in public facilities and spaces; supermarkets and food sources in the community; food waste reuse, redistribution and reduction; and support for communities. Pilot implementation of the tool in one local government demonstrated that the assessment process was feasible and likely to be helpful in guiding policy implementation.

**Conclusion:**

The Local Food-EPI+ tool and assessment process offer a comprehensive mechanism to assist local governments in benchmarking their actions to improve the healthiness, equity and environmental sustainability of food systems and prioritise action areas. Broad use of this tool will identify and promote leading practices, increase accountability for action and build capacity and collaborations.

The characteristics of contemporary food systems have a major influence on both population diets and environmental sustainability^(1)^. A key driver of unhealthy diets and high levels of diet-related disease is unhealthy food systems that are dominated by highly accessible, relatively cheap and heavily promoted unhealthy foods^(2)^. Many aspects of food systems also contribute to inequalities in diet quality and diet-related chronic diseases^(3,4)^. For example, in Australia, extensive unhealthy food marketing and the relative density of unhealthy food outlets means that food environments in the most disadvantaged areas are often less healthy than those in the least disadvantaged areas^(5,6)^. Moreover, current food systems contribute to over one-third of emitted man-made greenhouse gases^(7)^, climate change, biodiversity loss, land degradation, depletion of freshwater resources and water contamination^(7,8)^. Accordingly, there has been an increased focus on transitioning to more sustainable food systems that promote food security and good nutrition for all in such a way that the natural environment is positively or neutrally impacted considering both current and future generations^(9)^.

It is widely recognised that all levels of government have a critical role to play in improving the healthiness, equity and environmental sustainability of food systems^(10–12)^. Effective policy responses can improve the characteristics of food systems, that, in turn, can contribute to reductions of all forms of malnutrition (including undernutrition, overweight and obesity) and improve a community’s health^(13)^. Globally, government implementation of recommended policies in this area has generally been slow and inadequate^(10,14)^. This has led to an increased focus on accountability as a means of both monitoring policy progress and helping to drive policy change^(15)^. The International Network for Food and Obesity/noncommunicable diseases Research, Monitoring and Action Support (INFORMAS) is a global network (active in more than sixty countries) that aims to strengthen accountability by monitoring actions to create healthier food environments^(8)^. The Healthy Food Environment Policy Index (Food-EPI) tool, developed by INFORMAS and adopted in over fifty countries (including Australia^(16)^), has benchmarked government action for creating healthy food environments at mainly national but also some sub-national levels^(14,17)^. A first foray in using the Food-EPI tool and process to assess local government nutrition policy progress was conducted in three municipalities in Canada in 2018^(18)^, but the Food-EPI tool has not been applied at the local government level elsewhere. Globally, there are several other initiatives, including the *Milan Urban Food Pact* ^(19)^ and the *Barilla Foundation’s Food Sustainability Index* ^(20)^, that aim to support local governments in creating healthy and/or environmentally sustainable food systems by setting relevant standards based on indicators of good practice. Existing resources for local governments in the area either focus on a single component of food systems (such as agriculture, food losses and waste or nutritional challenges) or consider multiple aspects of the food supply chain but do not include processes and mechanisms to benchmark policy responses against good practice.

This paper focuses on local government policy action in the Australian context. The laws and regulations governing local governments in Australia vary by state; however, in all states, local governments share primary functions of local-level governance, planning, service delivery, community development, regulation and asset management^(21)^. As part of these functions, local governments can take a range of actions to improve the healthiness, equity and environmental sustainability of food systems and to support communities to have healthy and environmentally sustainable diets^(11,22,23)^. These actions include taking measures within their jurisdictions and through partnering, funding, coordinating and supporting policy action roles undertaken by other government and non-government actors^(22)^. A comprehensive, coherent policy response is likely to include actions across multiple areas, including land-use management, location and density of food outlets, food procurement, provision of resources to communities, education and initiatives that promote healthy and environmentally sustainable behaviours, restrictions on marketing of unhealthy food, and food waste minimisation strategies, amongst others^(11,12,24–26)^. In some Australian states, such as Victoria, local governments are required by state government public health laws to include both increasing healthy eating and tackling climate change in their Municipal Public Health and Wellbeing Plans^(27)^. However, there is currently large variation in the extent to which local governments implement recommended policies for improving the healthiness and environmental sustainability of food systems^(12,22,25)^. Furthermore, there is limited understanding of the most effective and equitable policy options available to local governments in this area^(12)^. In addition, many local governments experience internal barriers to policy adoption, including limited resources, limited leadership support and perceived lack of power to make change^(25–27)^.

This study aimed to: (1) develop a tool and assessment process to benchmark local governments in Victoria, Australia, on their implementation of policies to improve the healthiness, equity and environmental sustainability of food systems; and (2) assess the usefulness and feasibility of the tool and assessment process by implementing it within one Victorian local government. The longer-term goal is to apply the tool in local governments across Australia and globally, and, thereby, stimulate efforts to increase accountability for action at this level of government.

## Methods

### Scope and definitions

For the purposes of the study, ‘food systems’ were defined as all elements and activities that relate to production, processing, distribution, preparation, retail, consumption and disposal of food^(7)^. ‘Healthy and environmentally sustainable food environments’ were conceptualised as food environments in which: (a) foods and beverages that contribute to a healthy and environmentally sustainable diet are widely available, affordably priced and widely promoted; (b) foods and beverages that do not support healthy and environmentally sustainable diets are less readily available and are not promoted; and (c) the community is supported to adopt healthy and environmentally sustainable diets, including through the facilities, programmes and information made available.

Our conceptualisation of ‘healthy foods and beverages’ was based on the Australian Dietary Guidelines (ADG)^(28)^ and other relevant Australian state and territory government guidelines (e.g. the Victorian Government Healthy Choices guidelines^(29)^). For this study, healthy food and beverages correspond to those classified as part of the ‘five food groups’ (products recommended for regular consumption) as part of the ADG. These foods are generally lower in added sugar, sodium and harmful fats. We conceptualised healthy diets as being consistent with the recommendations of the ADG, including having limited consumption of unhealthy (‘discretionary’) foods that are often ‘ultra-processed’ and high in energy and added sugar, sodium and/or harmful fats. In operationalising the concept of ‘environmentally sustainable foods and beverages’, the focus was on reducing the environmental impact of foods and beverages, including through the way food is produced, minimising food waste and food packaging, preferencing fresh and minimally processed locally produced seasonal food, and limiting red meat^(7)^. The term ‘policy’ was used broadly to refer to all relevant government strategies, plans, regulations and related activities in a particular area.

### Development of the Local Food-EPI+ tool

The development of the Local Food Systems Policy Index (Local Food-EPI+) tool, including its domains, policy areas, indicator statements, scoring criteria, weightings and good practice examples, occurred over a 12-month period (July 2021–June 2022). An iterative approach was adopted, guided by regular input from an expert advisory committee consisting of local and international experts (*n* 35). These members included local government representatives and policy-makers (*n* 7), academic researchers (*n* 24) and health promotion practitioners (*n* 4). The expert advisory committee was established as part of the activities of the Nourish Network – a multi-sector collective working collaboratively to improve the health of food environments^(30)^.

Our starting point for the development of the Local-Food EPI+ tool was the domains and indicators used in the Food-EPI developed by INFORMAS^(31)^. All aspects of the Food-EPI tool were tailored to the local government context in Australia, with modifications and additions made to incorporate environmental sustainability and equity considerations, and an extension of the scope to include ‘food systems’ rather than the narrower concept of ‘food environments’. In the first instance, these changes were informed by the domains and indicators of the University Food Assessment (Uni-Food) tool developed by INFORMAS to benchmark the healthiness and environmental sustainability of university food environments in Australia^(8)^ and the indicators used in the *Milan Urban Food Policy Pact* ^(19)^. In consultation with the expert advisory committee, the tool was further refined based on academic literature, websites and policy documents available to the public on recommended local government actions to create healthy, equitable and environmentally sustainable food systems and improve population diets. This literature was identified based on searches of key academic databases (Web of Science, Scopus, Embase and Medline) using search terms such as ‘food system* sustainability’, ‘benchmark*’ and ‘policy’ or ‘policies’. Targeted searches of the websites of key relevant organisations were undertaken to identify relevant publicly available information (such as government reports, policy frameworks, sustainable food procurement templates, guides for local government action and food system consensus statements), as well as Internet searches using Google, guided and supplemented by the authors’ knowledge. The evidence was then grouped by domain and policy area and summarised for the expert panel to consider as part of the process of developing and refining the indicators.

Draft indicators (and related statements of good practice) were provisionally selected for inclusion if they were recommended by the expert advisory committee as likely to be effective based on local government responsibilities for different aspects of food systems and were applicable to the local government context in Victoria. The draft indicators were iteratively reviewed by the expert advisory committee, including refinements to avoid overlap, add specificity for the local context and increase local relevance. There were multiple rounds of review, including a combination of workshops and written feedback provided to the research team over a period of 4 months. Discussions with the committee continued until all concerns were addressed to their satisfaction. Indicators were grouped into policy areas and domains (general topic areas) on advice of the committee. For each indicator, where available, good practice examples by local governments in Australia and globally were identified from the literature and on advice from the committee.

The Local Food-EPI+ tool was designed to allocate local governments with a ten-point scale score for each indicator, based on the extent of policy implementation compared with the statement of good practice (the assessment process is described further below). The relative weightings for each policy area and domain were developed through input from the expert advisory committee. This input was provided initially via an online survey where each member assessed the relative importance of each domain and policy area and assigned a weighting for each (out of possible 100 points in total). The survey results were collated, with final weightings for each component determined by consensus discussions with the expert advisory committee.

### Adaptation of Food-EPI assessment process to the local government setting

The process for applying the Local Food-EPI+ tool was modelled on the Food-EPI assessment process^(31)^. In consultation with the expert advisory committee, the Food-EPI assessment process was adapted to the Australian local government context and designed for repeated self-assessment (approximately every 2 to 3 years) by local governments to measure progress over time. The process was adapted to include provision for the incorporation of audit data related to the characteristics of different aspects of food systems (e.g. the extent of food marketing in different settings) as part of the assessment process.

**Fig. 1:**
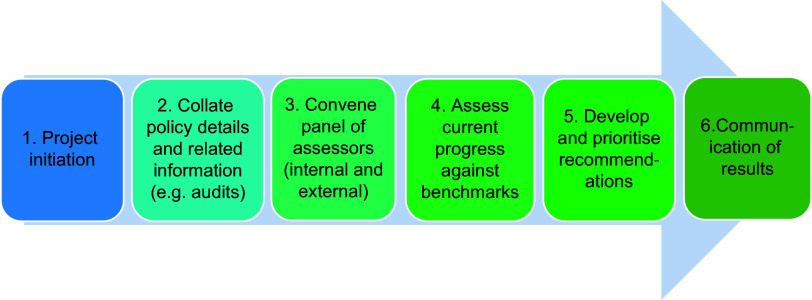
Process for assessment using the Local Food Systems Policy Index (Local Food-EPI+) tool

The six-step process for assessing local governments using the Local Food-EPI+ tool is shown in Fig. 1. The required inputs to the process, activities, expected outputs and desired outcomes (short term (6–12 months), medium term (1–3 years) and long term (>3 years)) are included in the programme logic model (as outlined in Online Supplementary Information, Appendix 1).

Step one involves project initiation, including securing senior-level support for the project in the local government being assessed, establishing a project team and associated resources, determining a timeline, and adjusting the tool and process for relevance to the context of the local government being assessed. In this step, particular indicators may be identified as not relevant to the context, for example, for a local government in a metropolitan region, some of the indicators focused on commercial agricultural land may not be relevant. Particular indicators may also be identified as not applicable based on a local government’s jurisdiction to act. For example, in Australia (unlike cities in the USA and elsewhere), local governments do not have the jurisdiction to tax unhealthy foods, and so indicators related to implementation of food taxes would not be applicable to the local government context in Australia. In these instances, it may be deemed appropriate to change the focus of a particular indicator to reflect actions that are within the jurisdiction of the local government within a policy area. For example, in the policy area of food taxes, there may be a role for local governments to advocate to other levels of government to implement taxes, and the relevant indicator may be altered to reflect such a role.

Step two involves collating policy details for the local government for each indicator in the Local Food-EPI+ tool. The process typically requires consultation with multiple teams within local government working in relevant areas, such as health promotion, food safety and urban planning. This step of the process also provides for the identification of existing monitoring or audit data and/or the collection of new primary data on the characteristics of local food systems. The inclusion of these data informs assessment of policy gaps and the extent to which relevant policies are implemented in practice. Examples of audit data that could be included are as follows: the proportion of healthy food and beverages available in government-owned/managed settings; the proportion of reusable, recyclable or biodegradable food packaging used in food retail outlets; the proportion of outdoor advertisements with unhealthy food and beverage messaging; and the prevalence of free drinking water in public spaces.

Step three involves convening a panel of assessors. The panel could include representatives from the local government being assessed, from other local governments, health promotion and sustainability practitioners, and public health and environmental sustainability researchers. A key adaptation for the Local Food-EPI+ was the provision for local government officials to be part of the assessment panel, rather than the rating of government policies being conducted only by those external to government (as per the application of the Food-EPI tool in most of the other contexts in which it has been implemented). This move to incorporate aspects of self-assessment was made with a view to increasing the reach of the tool and the sustainability of its repeated application in a range of contexts, whilst also increasing potential impact by directly engaging those who are responsible for policy decisions as part of the assessment process.

Step four involves assessment of current progress against the good practice statements of the Local Food-EPI+ tool for each indicator, based on the policy details of the local government concerned (and associated monitoring/audit data if relevant). The assessment panel members each allocate a score out of 10 for each indicator. In line with the approach used in the Food-EPI tool, the assessment criteria consider both the ‘quality’ of the policy action and the ‘extent’ of policy implementation. ‘Quality’ includes how comprehensive the policies are compared with the statement of good practice for that indicator, whether all aspects of the indicator are incorporated, and the effectiveness or likely effectiveness of the policy actions. ‘Extent’ of implementation is with reference to the notional policy cycle that extends from agenda setting and initiation, through policy development and implementation, to monitoring and evaluation^(31)^. Median scores for each policy area and domain are averaged to derive a domain score (as a percentage out of 100). An overall score (as a percentage out of 100) for the local government is calculated based on the policy area and domains scores, weighted based on their relative importance.

Step five involves developing and prioritising recommendations specific to the local government, based on the results of step four, and with reference to the good practice statements.

Step six centres on communication of the results to key stakeholders, including executive and senior managers in local government. This knowledge exchange may take the form of a report that presents performance in each domain, highlights key strengths and areas of good practice and identifies the full set of recommendations, including short-term priorities. In the future, as more local governments are assessed using the tool, communication could also include detailed comparison to other local governments and progress over time. Such comparisons are likely to be most relevant for local governments operating in similar contexts, for example, within the same country and with similar demographic characteristics.

### Pilot implementation of the Local Food-EPI+ tool and process

In 2022, a pilot test of the Local Food-EPI+ tool and process was conducted in one regional Victorian local government, the City of Greater Bendigo (CoGB). The pilot allowed for feasibility testing and refining of the tool and assessment process to inform future broader application of the tool.

Data related to policy implementation for each indicator in the Local Food-EPI+ tool were collected by a government official from CoGB over a 5-month period, in conjunction with the research team. The process of collecting policy information entailed extensive involvement with other officials from CoGB, including from local government areas of waste management, agriculture, climate change and environment, and town planning.

To supplement policy information and to inform assessment of the extent of implementation of policies, data on the characteristics of selected aspects of food systems in CoGB were also collected (food systems audit). Areas of focus for the food systems audit were determined in conjunction with CoGB based on the data they already had available (only data related to the last 3 years were considered), the feasibility of new data collection and the perceived importance of the topic area in the CoGB context. Collection of new data focused exclusively on describing food environments, including advertising present at sports grounds, foods available in sport and recreation centres, and availability of water taps/fountains in public areas. These data were collected by visual inspection of local government-owned or managed facilities, including food retail displays and menus, vending machines, food promotion activities, waste monitoring practices, serving ware use and drinking tap or fountain availability. For these data collection activities, we utilised the Uni-Food food environment assessment tool, adapted to suit the indicators in the Local Food-EPI+ tool^(8)^. Data were collected from seventy-nine facilities by health science students who were trained by the research team in the use of the tool.

The CoGB panel assessment was conducted as an online half-day workshop (held in August 2022) with a range of experts in the field (assessment panel, *n* 28). Assessment panel members included officials from CoGB (*n* 3), a representative from the Victorian Local Governance Association (*n* 1), representatives from other local governments in Victoria (*n* 15) and academic researchers (*n* 9). The areas of expertise of the assessment panel members were predominantly in nutrition/health promotion (*n* 20) and environmental sustainability (*n* 8). Panel members entered their assessments using a Research Electronic Data Capture browser-based online survey, as part of the facilitated workshop. The research team collated the assessment scores and conducted an inter-rater reliability test using Gwets AC2 in R studio (V4.1.2).

Based on the assessment results, the research team developed a list of recommendations for CoGB, in consultation with an official from CoGB. Assessment panel members were subsequently invited to complete a further online survey (October 2022) to prioritise the list of recommendations for action in the short term (next 2–3 years). The results of the prioritisation survey were discussed with the official from CoGB and used to identify priority recommendations, taking into account the local context, strategic priorities, operational requirements and feasibility of implementation. The assessment panel members also identified ‘quick wins’ – those recommendations considered highly feasible to implement in the short term. The assessment results and recommendations were collated by the research team into a report. This report was presented (December 2022) to CoGB senior decision-makers, including the CEO and senior managers, the Mayor, local councillors and other key stakeholders.

We conducted a limited evaluation of the Local Food-EPI+ implementation process in the CoGB that focused on the views of assessment panel members. For these purposes, after the completion of the prioritisation survey (October 2022), we invited assessment panel members to complete an online survey that examined their experiences of the assessment process, their thoughts on the value of the Local Food-EPI+ tool, including its usability, and the knowledge and connections gained, if any (see Online Supplementary Information, Appendix 2 for the evaluation questionnaire). Responses to quantitative questions were analysed using descriptive statistics, and qualitative responses (to open ended questions) were summarised.

An ethics application for the study was approved by the Deakin University Human Ethics Advisory Group (Reference HEAG-H 62_2022).

## Results

### Local food-EPI+ tool

The Local Food-EPI+ tool consists of sixty-one indicators in twenty-five policy areas, across ten policy domains. The domains include: leadership; governance and platforms for engagement; funding and resources; monitoring and intelligence; food production and supply chain; food promotion; food provision and retail in council facilities and public spaces; supermarkets and food sources in the community; food waste reuse, redistribution and reduction; and support for communities (see Table 1). The ‘Leadership’ (15 %) and ‘Food provision and retail in council facilities and public spaces’ (12 %) domains were weighted most heavily (see Table 2). Further details of each domain, policy area and indicators of the Local Food-EPI+ tool are in Online Supplementary Information, Appendix 3. Illustrative examples of good practice in relation to each policy area are provided in Online Supplementary Information, Appendix 4.

**Table 1:** Domains and policy areas (with associated weightings) and indicators as part of the Local Food Systems Policy Index (Local Food-EPI+) tool. Refer to Online Supplementary Information, Appendix 3 for a full description of the indicators

Domain	Domain weight	Policy area	Policy area weight	Indicator
Leadership	15 %	**LEAD1**: Strategy and implementation plans for healthy, equitable and environmentally sustainable food systems and improving population nutrition	100 %	**LEAD1·1** Strong, visible high-level support **LEAD1·2** Over-arching goals in place **LEAD1·3** Detailed strategy **LEAD1·4** Comprehensive implementation plan **LEAD1·5** Explicit priority to reducing diet-related health inequalities
Governance and platforms for engagement	10 %	**GOVER1**: Structures and platforms for collaboration, engagement and cohesion	80 %	**GOVER1·1** Collaboration between council departments **GOVER1·2** Actively participate in relevant networks **GOVER1·3** Systems promote transparent communication and engage the community **GOVER1·4** Identify and manage conflicts of interest
		**GOVER2:** Use of evidence to inform policy	20 %	**GOVER2·1** Use of evidence, including routine evaluation
Funding and resources	10 %	**FUND1**: Government workforce to create healthy, equitable and environmentally sustainable food systems and improve population nutrition	50 %	**FUND1·1** Sufficient capacity (number of staff and their capabilities) dedicated
		**FUND2:** Funding to create healthy, equitable and environmentally sustainable food systems and improve population nutrition	50 %	**FUND2·1** Sustained (‘core’) funding to support efforts **FUND2·2** Sufficient funding to promote and support community-led initiatives
Monitoring and intelligence	10 %	**MONIT1**: Monitoring of local food systems	70 %	**MONIT1·1** Relative density of healthy food outlets *v*. unhealthy food outlets **MONIT1·2** Price and affordability of culturally appropriate baskets of healthy (compared with unhealthy) foods and beverages **MONIT1·3** Availability of healthy (compared with unhealthy) food and beverages **MONIT1·4** Characteristics of food advertising (e.g. healthy *v*. unhealthy ads) and sponsorship **MONIT1·5** Levels of food waste **MONIT1·6** Use of unsustainable food packaging and/or single-use plastics
		**MONIT2:** Monitoring of population diets, food system environmental sustainability and related health outcomes	30 %	**MONIT2·1** Population food and nutrient intake **MONIT2·2** Population health outcomes related to nutrition
Food production and supply chain	10 %	**PRODS1**: Sustainable food production and land management	75 %	**PRODS1·1** Sustainable land management practices ensure natural resources are protected and enhanced, and sustainable farming is promoted **PRODS1·2** Protect agricultural land **PRODS1·3** Producers, processors, food retailers and caterers are trained and supported on ways to improve energy, water and other resource efficiency across the food supply chain
		**PRODS2:** Support for urban agriculture	25 %	**PRODS2·1** Support urban agriculture on council owned or managed land
Food promotion	10 %	**PROMO1:** Protection of children from exposure to unhealthy promotion activities	75 %	**PROMO1·1** Restrict the exposure of children (including adolescents) to the promotion of unhealthy foods and beverages and related brands in council owned/managed settings **(non-retail)**
		**PROMO2:** Healthy sport sponsorship	25 %	**PROMO2·1** Eliminate sponsorship from brands related to unhealthy foods and beverages **PROMO2·2** Restrict sponsorship from brands related to unhealthy foods and beverages, including tailored support for culturally diverse communities
Food provision and retail in public facilities and spaces	12 %	**PROV1:** Healthy and environmentally sustainable food procurement, provision and catering	80 %	**PROV1·1** Targets for the proportion of food and beverage procured by council that is healthy and environmentally sustainable **PROV1·2** Food and beverage procurement activities contribute to improvements in the environmental sustainability of food supply chains **PROV1·3** Food policy for **food outlets operating in council owned/managed facilities**, including criteria related to availability, accessibility, affordability and promotion **PROV1·4** Food policy related to **foods and beverages provided/sold at council owned/managed community events**, including criteria related to availability, accessibility, affordability and promotion **PROV1·5** Food policy related for **internal food and beverage provision** that applies in all council owned/managed facilities, including criteria related to availability, accessibility, affordability and promotion **PROV1·6** Healthy and environmentally sustainable food and beverage provision and catering in council owned/managed facilities and events, including tailored support for culturally diverse communities
		**PROV2:** Access to free drinking water	20 %	**PROV2·1** Accessibility of free drinking water in public spaces and venues
Supermarkets and food sources in the community	8 %	**RETAIL1:** Availability and accessibility of healthy and environmentally sustainable food and beverage retail outlets	30 %	**RETAIL1·1** Availability and accessibility of healthy and environmentally sustainable food and beverage retail outlets **RETAIL1·2** Local food producers to link to local food procurement activity, hospitality businesses, farmers’ markets and consumers **RETAIL1·3** Public transport/active transport options linking healthy food retail outlets and residential areas **RETAIL1·4** New residential developments are built close to food retail outlets selling healthy and environmentally sustainable food **RETAIL1·5** Advocate for changes to the State Planning Policy framework
		**RETAIL2:** Support for supermarkets and other grocery outlets to improve the healthiness of their in-store environments	20 %	**RETAIL2·1** Work with supermarkets and other grocery outlets to incentivise their customers to purchase and consume healthy food and disincentivise unhealthy options
		**RETAIL3:** Support for food retail outlets (non-grocery) to improve the healthiness of their in-store environments	20 %	**RETAIL3·1** Work with food retail outlets (non-grocery) to improve the in-store availability, affordability and promotion of healthy foods and reduce the availability and promotion of unhealthy foods
		**RETAIL4:** Support for provision of point-of-purchase nutrition and environmental sustainability information	15 %	**RETAIL4·1** Provision of simple, easy-to-understand nutrition-related information at point of purchase **RETAIL4·2** Provision of simple, easy-to-understand environmental sustainability-related information at point of purchase
		**RETAIL5:** Reduction/reuse of unsustainable packaging materials	15 %	**RETAIL5·1** Use of environmentally sustainable packaging of food products and/or promote minimal use of packaging materials **RETAIL5·2** Food retailers to encourage the use of ‘Bring Your Own’ (BYO) or returnable packaging
Food waste reuse, redistribution and reduction	5 %	**REDUC1:** Food waste reuse, redistribution and reduction	100 %	**REDUC1·1 Council internal operations** to prevent and minimise food waste going to landfill **REDUC1·2** Coordinated redistribution and/or donation of healthy food to prevent commercial food waste **REDUC1·3** Educate and support the community to prevent and minimise food waste
Support for communities	10 %	**COMM1:** Coordinated support for community initiatives to promote healthy, equitable and environmentally sustainable food systems and diets	15 %	**COMM1·1** Community initiatives to create and maintain healthy, equitable and environmentally sustainable food systems
		**COMM2:** Food relief services	20 %	**COMM2·1** Nutritious and affordable social meal provision programmes **COMM2·2** Nutrition-related training, support and/or advice to organisations that provide food relief services **COMM2·3** Financial support and/or subsidies provided to food relief organisations includes considerations of culturally appropriate healthy and environmentally sustainable food
		**COMM3:** Breast-feeding policies, programmes and facilities	20 %	**COMM3·1** Supportive environment is provided for people who breastfeed and/or express milk **COMM3·2** Focused education to support new parents achieve optimal infant feeding practices **COMM3·3** Public education to promote, support and protect breast-feeding and the right to breastfeed infants in public
		**COMM4:** Provision of community facilities for cooking and food preparation	15 %	**COMM4·1** Communal infrastructure related to food preparation **COMM4·2** Adequate food storage and preparation areas for healthy cooking
		**COMM5:** Engagement and training for public and private sector organisations	15 %	**COMM5·1** Workplaces to provide healthy food and beverage environments and promote healthy and environmentally sustainable behaviours
		**COMM6:** Social marketing campaigns related to healthy and environmentally sustainable food systems and improving population nutrition	15 %	**COMM6·1** Social marketing to promote healthy and culturally appropriate environmentally sustainable foods and diets **COMM6·2** Social marketing, across a range of media on reducing/discouraging single-use plastics and plastic packaging at the point of sale, pick-up and delivery of food

Environmental sustainability was incorporated in a number of parts of the Local Food-EPI+ tool. First, the standard Food-EPI tool was expanded to include additional domains and policy areas, including in the areas of ‘food waste reuse, redistribution and reduction’ and ‘food production’. In addition, new indicators were added and several indicators were expanded in scope to include consideration of environmental sustainability. For example, in the ‘food provision’ domain, an indicator related to ‘food procurement’ included consideration of ways in which procurement activities could contribute to improvements in the environmental sustainability of food systems, drawing on examples of what councils are achieving in this area. For example, Mornington Peninsula Shire Council’s draft ‘Catering Contract’ criteria included sustainability targets for at least 30 % of food provided to be free from animal-sourced proteins. In the ‘supermarkets and food sources in the community’ domain, an indicator was added relating to supporting the provision of simple, easy-to-understand environmental sustainability-related information at point of purchase.

While equity features in many indicators of the standard Food-EPI tool, we made several modifications to more explicitly include equity considerations in the Local-Food EPI+ tool. For example, in the ‘supermarkets and food sources in the community’ domain, one of the indicators focuses on policies and/or programmes that encourage the availability and accessibility of retail outlets selling fresh fruit and vegetables, with a particular focus on low-income neighbourhoods. In addition, several indicators related to the provision of resources or guidelines to support communities included specific mention of the need for tailored support for culturally diverse communities.

### Pilot application of the Local Food-EPI tool+ in the City of Greater Bendigo

**Fig. 2:**
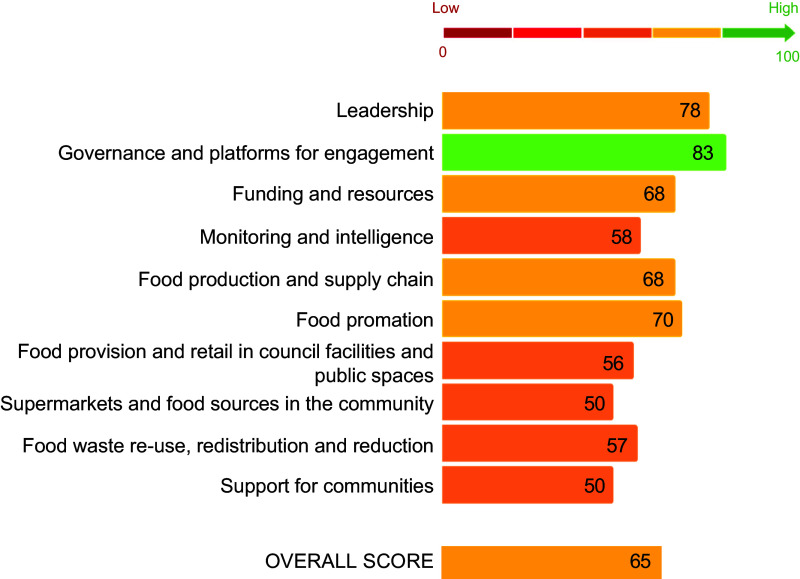
Results from the pilot assessment (score out 100) of the implementation of policies for creating healthier, equitable an environmentally sustainable food systems in the City of Greater Bendigo using the Local Food Systems Policy Index (Local Food-EPI+) tool in 2022. ‘High’ (81–100 %); ‘very good’ (61–80 %); ‘good’ (41–60 %); ‘fair’ (21–40 %); and ‘very low, if any’ (0–20 %) implementation

The pilot implementation of the Local Food-EPI+ tool was completed in CoGB in 2022. All twenty-eight members of the assessment panel completed the assessment of each indicator. The CoGB received an overall score of 65 % (Fig. 2). CoGB performed best in the ‘governance and platforms for engagement’ (83 %) and ‘leadership’ domains (78 %). CoGB scored lowest in the ‘support for communities’ (50 %) and the ‘supermarkets and food sources in the community’ (50 %) domains. In the ‘governance and platforms for change’ domain, the strong collaboration between council departments, transparent communication and engagement with the community, and active participation in relevant networks contributed to their strong performance. Another area of strong performance was in the ‘funding and resources’ domain, where they scored well in the ‘government workforce’ policy area, reflecting the city’s high-level capability with three staff dedicated to food systems work.

The reliability of the tool was assessed by inspecting inter-rater reliability of the assessment scores. The Gwet’s AC2 inter-rater reliability coefficient was 0·82 (82 % CI 0·81, 0·84). The policy details and audit results for CoGB are available on request.

Twenty-one recommendations were developed for the CoGB, spanning all domains of the Local Food-EPI+ tool. The shortlist of prioritised recommendations for action in the short term (next 2 to 3 years) included: (1) introducing over-arching goals and adopting specific targets for creating and maintaining healthy, equitable and environmentally sustainable food systems, improving population nutrition, and preventing diet-related diseases; (2) allocating an ongoing budget and increasing funding to lead projects and support efforts to create and maintain healthy and environmentally sustainable food systems; and (3) ensuring healthy and environmentally sustainable food procurement. The ‘quick wins’ were as follows: (1) implementing a policy to restrict unhealthy food promotion in local government-owned/managed settings where children gather; (2) progress policy to improve the availability of freshwater in public spaces and venues; (3) expanding efforts working with supermarkets and food retail outlets to link healthy food retail outlets and residential areas; and (4) supporting provision of simple, easy-to-understand nutrition-related information at point of purchase with consideration of cultural diversity. Refer to Table 2 for the full set of recommendations, and see Online Supplementary Infortmation, Appendix 5 for excerpts from the report provided to CoGB.

**Table 2:** Recommendations for the City of Greater Bendigo for creating healthier, equitable an environmentally sustainable food systems, from the pilot application of the Local Food Systems Policy Index (Local Food-EPI+) tool and process, 2022

Domain	Policy recommendation
Leadership	Introduce over-arching goals (specific, measurable and time-bound) for creating and maintaining healthy, equitable and environmentally sustainable food systems, improving population nutrition and preventing diet-related diseases.*
Funding and resources	Allocate ongoing budget for local government to lead projects and initiatives to support efforts to create and maintain healthy and environmentally sustainable food systems.*
	Increase funding to support community-led initiatives for creating healthy, equitable and environmentally sustainable food systems, improving population nutrition and preventing diet-related diseases.*
Monitoring and intelligence†	Routinely (e.g. every 2 years) monitor and report on the affordability of healthy (compared with unhealthy) food and beverages, extending previous monitoring efforts in this area (e.g. applying a standardised Healthy Food Basket Survey using the ASAP tool^(32)^). Routinely (e.g. every 2 years) monitor and report on the characteristics of food advertising and sponsorship in the community, expanding on initial monitoring conducted in 2022 (e.g. with the assistance of university students on placement with council). Routinely (e.g. every 2 years) monitor and report on the relative density of healthy food outlets *v*. unhealthy food outlets, by geographic area, building on participation in the Australian Food Retail Environment Monitoring Tool Victoria study in 2022. Routinely assess, report and analyse population health outcomes related to nutrition, population food and nutrient intake, using existing datasets (e.g. state-based) where possible.
Food production and supply chain	Provide resources and/or guidelines to ensure producers, processors, food retailers and caterers are trained and supported on ways to improve energy, water and other resources efficiency across the food supply chain, including tailored support for culturally diverse communities. Support urban agriculture on local government-owned or managed land by continuing and building on efforts to develop and implement the Community Gardens Policy and the Nature Strip Policy.
Food promotion	Implement the Healthy Facilities Policy to restrict the exposure of children (including adolescents) to the promotion of unhealthy foods and beverages and related brands in local government-owned/managed settings.‡
	Include contractual obligations for all organisations, such as community groups and sports clubs, that use local government facilities and/or obtain funds from local government, to eliminate sponsorship from brands related to unhealthy foods and beverages and to restrict the provision of unhealthy foods and beverages, whilst supporting provision of healthy options.
Food provision and retail in council facilities and public spaces	Develop and adopt specific targets for the proportion of food and beverage procured by local government (across all relevant operations) that is healthy and environmentally sustainable (e.g. add criteria within the catering contract that includes sustainability targets for at least 30 % of food provided to be free from animal-sourced proteins).*
	Ensure healthy and environmentally sustainable food at community events, by developing and implementing a clear, consistent food provision policy including criteria related to availability, accessibility, affordability and promotion.*
	Improve availability of freshwater by continuing to progress the ‘Drinking water fountains’ project to establish prioritisation criteria to decide which areas are most in need of and suitable for water fountain installation.‡
Supermarkets and food sources in the community	Ensure that there are public transport/active transport options linking healthy food retail outlets and residential areas by implementing the 10-year Walking Cycling Strategy.‡
	Expand the Greater Bendigo Healthy Catering Guide to apply to in-store menus to expand efforts to improve the in-store availability, affordability and promotion of healthy foods and reduce the availability and promotion of unhealthy foods. Continue and expand efforts of the Healthy Sports Club project (HHV) to develop and implement programmes that support provision of simple, easy-to-understand nutrition-related information at point of purchase, including consideration for culturally diverse communities.‡
	Develop and implement programmes that support provision of simple, easy-to-understand environmental sustainability-related information at point of purchase, including consideration for culturally diverse communities.
Support for communities	Include contractual obligation that any financial support provided to food relief organisations includes considerations of culturally appropriate, healthy and environmentally sustainable food, accompanied by nutrition-related training, support and/or advice to relevant organisations (drawing on relevant state policies and/or guidelines where possible). Develop and implement public education to promote, support and protect breast-feeding and the right to breastfeed infants in public, including tailored support for culturally diverse communities. Commit to ongoing, long-term support for social marketing campaigns, developed based on best-practice principles, as part of broader efforts to improve population health and promote healthy and environmentally sustainable diets, including tailored support for culturally diverse communities.

* Priority recommendations for action in the short term (2–3 years).

† Monitoring does not need to be conducted by the council; data can be drawn from other sources if available.

‡ ‘Quick wins’ (considered highly feasible for short-term implementation).

### Evaluation of the benchmarking process

An evaluation survey was completed by twenty of the assessors (response rate 71 %) – refer to Online Supplementary Inforrmation, Appendix 6 for a summary of the quantitative survey results. All respondents (100 % agreed or strongly agreed) indicated that their participation in the assessment process led to an increase in their knowledge of strategies to increase both the healthiness and environmental sustainability of food systems at the local government level. CoGB staff who participated in the assessment process reported that the tool would be useful to their work by way of helping guide development of internal procedures and policies. Local government respondents highlighted that their participation in the assessment process was particularly valuable in enabling them to identify examples of policies that could be implemented locally.

Respondents indicated that they found the assessment process to be either ‘easy’ or ‘fairly easy’ to complete (seventeen out of twenty respondents). They indicated that they had a clear understanding of the scoring procedure. Many respondents described the scoring process as succinct, and they reflected that there was sufficient information to conduct the scoring confidently. One respondent observed that the ease in scoring each indicator was dependant on the assessor’s experience and/or knowledge in this area.

A small number of respondents expressed reservations regarding the potential scalability of the tool. While these respondents indicated that they believed the tool was an effective benchmarking tool, they indicated that many local governments were under-resourced and would find it challenging to find the time and resources to implement the tool without dedicated support for doing so:

*‘It’s a huge amount of work for a local government to self-assess their progress, particularly if they’re small, have limited resourcing, or limited interest in food systems’. [Respondent, academic researcher]*

Conversely, many councils liked the comprehensiveness of the tool and best practice examples:

*‘[The tool provides] a very comprehensive systems approach with clear indicators. Such a large assessment group with representation across the system will make for a robust assessment’. [Respondent, council member]*

## Discussion

This study developed the Local Food-EPI+ tool and assessment process to benchmark the implementation of local government policies for creating healthy, equitable and environmentally sustainable food systems and successfully piloted the tool in one Victorian local government. The Local Food-EPI+ tool comprises sixty-one indicators across ten domains and is designed for self-assessment by local government officials. The Local Food-EPI+ tool represents the first food policy benchmarking tool to include health and environmental sustainability tailored to the local government level.

The Local Food-EPI+ tool and assessment process were modelled on the well-established Food-EPI tool developed by INFORMAS^(31)^. The standard Food-EPI tool was adapted to the local context and expanded to include environmental sustainability and to explicitly include an equity focus. Several domains and policy areas from the standard Food-EPI tool (e.g. in the areas of ‘food labelling’ and ‘food pricing’) were considered less relevant for local governments in the Australian context due to jurisdictional limitations. Such adjustments to the Food-EPI tool for the local context are consistent with changes made to the Food-EPI tool when it was implemented in three Canadian municipalities in 2018.

The Local Food-EPI+ tool builds on existing tools and frameworks, such as the *Milan Urban Food Pact*, *Food Action Cities* and the *Glasgow Food and Climate Declaration*, to help cities share actions to shape their food environments for improved health and/or environmental sustainability^(19,33,34)^. Unlike the Local Food-EPI+ tool, existing tools and frameworks either focus on both aspects of improving health and environmental sustainability but do not include any benchmarking, or they focus on either health or sustainability elements. For example, the Centre for Food Policy at the University of London recently prepared a guide of forty-five actions for policy-makers to shift food systems towards environmental sustainability, but their guide does not include a specified process of measuring implementation or conducting benchmarking^(35)^. In contrast, the South Australian Local Government guide *Creating Healthier Local Food Environments* ^(36)^ is designed to assist local governments in assessing implementation of current policies, plans and actions in improving their food environments but does not comprehensively include environmental sustainability considerations. Similarly, the Centre for eResearch and Digital Innovation *How Well Are We Adapting Tool* is focused on assisting local governments to monitor, evaluate and report on climate adaptation only, with limited consideration of nutrition^(37)^. Accordingly, the strengths of the Local Food-EPI+ tool include: its comprehensive focus on health, equity and environmental sustainability; assessment of policy implementation through the policy stages of development, implementation, monitoring and evaluation; a process of benchmarking and assessment that is highly participatory; and policy recommendations that are customised to the needs of the local government concerned.

The pilot application in one Victorian local government demonstrated that implementation of the Local Food-EPI+ tool is feasible, with post-implementation evaluation by the assessment panel showing that it was likely to be viewed as valuable in helping local governments to develop an understanding of food-system-related policies and best practices and to assess their own policies and prioritise actions. Unlike the way in which the Food-EPI process has been implemented in most other settings (where assessment has typically been conducted independently of government), our pilot application of the tool in the CoGB included representatives of the CoGB as part of the assessment panel. The potential benefits of including local government staff as part of the assessment process are increased engagement, increased knowledge of best practice amongst council staff and increased opportunity for collaboration with relevant stakeholders^(38,39)^. Based on our experience in implementing the tool in Victoria, it is also likely to be easier to recruit local government staff to participate in the process, rather than recruiting externally. These potential benefits need to be balanced against the potential risk of bias resulting from self-assessment. We found that the CoGB staff that participated in the assessment panel rated each indicator similarly to the other (independent) participants in the assessment process. These findings contrast with those from Thailand where government participants rated policy progress higher than non-government participants as part of the Food-EPI assessment process at the national level^(40)^. The results from our study give us confidence that the planned self-assessment process for implementation of the Local Food-EPI+ tool is likely to prove reliable, but broader implementation and testing in other contexts is needed to confirm this. If external participants are used as part of the assessment process, it is recommended that food industry stakeholders are excluded due to potential financial conflicts of interest.

The evaluation of the pilot application nevertheless raised concerns regarding the level of resources required to implement the Local Food-EPI+ process, particularly in contexts where many local governments are under-resourced and face multiple competing priorities. The extent of data on the characteristics of local food environments to supplement the policy analysis will depend on the feasibility of local-level data collection and the availability of data from routine data collection processes and other sources. In the case of CoGB, the council had recently participated in an Australian Food Retail Environment Monitoring study^(41)^ that proved beneficial in providing information on the density per 10 000 population for each food outlet type classified. CoGB had also previously invested in monitoring of other aspects of their local food environments, including the price of food. It is recognised that not all local governments would have these data available. Moreover, the comprehensive scope of the tool and the related recommendations to come out of the process may be perceived by some stakeholders as too broad. Accordingly, there may be an appetite, in certain contexts, for applying only certain domains or policy areas, or implementing a slimmed down version of the tool. It will be important to assess the feasibility and utility of such approaches, and how aspects of the tool might be applied in different contexts and for different purposes. For example, one ongoing trial in Australia is examining changes in the healthiness of food service in sport centres, with plans to apply only selected domains of the Local Food-EPI+ tool as part of the evaluation of the study^(42)^. Broad application of the Local Food-EPI+ tool is likely to require the provision of training and resources (e.g. by state governments, health promotion organisations or researchers) to assist local governments with the Local Food-EPI+ process, including support for data collection and reporting of results. Furthermore, the templates, examples and processes developed as part of this study are likely to prove useful in supporting implementation of the Local Food-EPI+ tool in other contexts and in generating impetus to conduct additional monitoring of food environments.

While the Local Food-EPI+ tool has been developed for the Victorian context, we anticipate that the tool can be adapted for use by local governments in other Australian states and other countries. Application of the tool in different contexts will require consideration of the policy landscape and jurisdictional authority of local governments in different settings. For example, in countries where municipalities are responsible for childcare and school food environments, indicators may be added relating to efforts to create healthy food environments in these settings. Similarly, within different regions of Victoria, the Local Food-EPI+ tool may need to be tailored for differences such as municipality size or degree of regionality (e.g. rural *v*. metropolitan). For example, indicators for developing policies to protect agricultural land would be less relevant for a local government situated in a metropolitan region with limited agricultural land. The modification of the Local Food-EPI+ tool to different contexts may slightly limit comparability of assessments. However, as with the standard Food-EPI tool and other similar benchmarking initiatives (such as food company comparisons)^(43)^, the primary value of the tool is likely to be found in the process of benchmarking against good practice, rather than comparison across jurisdictions^(39)^. We recommend that the tool be adapted to fit the local context to maximise local impact and that those implementing the tool modify it (e.g. by adding or omitting particular indicators) to their context. Future assessment of additional local governments – within Victoria, across all Australian states, and internationally – would assist in identifying further examples of best practice and would enable some comparison between local governments and learning between them. It is also likely to build capacity and collaborations between local governments and other relevant stakeholders. While we have identified some examples of good practice for each indicator in the Local Food-EPI+ tool, practical examples of good practice across several of the indicators are still nascent. Furthermore, some of the good practice statements in the Local Food-EPI+ tool are deliberately defined in broad terms, to acknowledge that evidence-based examples of implementation are still emerging and to provide scope for local innovation. For example, in the policy area of ‘breast-feeding and infant feeding’, the tool does not specify which specific programmes to implement, rather noting that programmes should be implemented based on the best available evidence and the local context, and that activities in this area should be evaluated.

There is increasing evidence that highlights the barriers many local governments face when developing and implementing food policies and actions^(25,26)^. Resources, skills, knowledge, leadership support and food system literacy vary between local governments, and there is often a lack of communication and collaboration between departments^(25,26)^. The Local Food-EPI+ tool aims to encourage local governments to increase their involvement in food systems by engaging senior leadership and multiple departments as part of the assessment process, clearly illustrating the range and nature of possible actions, and providing practical examples of actions performed by other local governments. We expect further implementation of the tool to increase knowledge of good practice and knowledge sharing between local government representatives and external stakeholders, as well as increased engagement, collaboration and alignment to promote healthy, equitable and environmentally sustainable food systems^(38,39)^.

Alongside strategies at other levels of government and within other sectors, future widespread uptake of the Local Food-EPI+ tool could contribute to positive human health and environmental outcomes, including improvements to dietary quality and subsequent reductions in the prevalence of all forms of malnutrition and diet-related diseases, more localised food production and reductions in food-related greenhouse gas emissions^(39)^.

### Strengths and limitations

The development of the Local Food-EPI+ tool and process was based on the well-established Food-EPI tool and process, which has been widely implemented in Australia and globally. The tool was modified for the Australian local government context via an iterative approach based on best available evidence and using an expert advisory committee. A key strength of the Local Food-EPI+ tool is that it takes a holistic approach to improving food systems, moving beyond the health-related aspects of food systems to explicitly incorporate both environmental sustainability and equity. The Local Food-EPI+ assessment process is designed to be highly participatory, with recommendations developed, adapted and prioritised in conjunction with the council, which is likely to boost the feasibility of the policy recommendations. The tool incorporates self-assessment to facilitate broadscale uptake, with modifications to suit the local context.

A limitation of the study is that the Local Food-EPI+ tool was piloted in only one local government. Broader implementation within local governments of varying size and location is needed to examine feasibility of implementation in different local jurisdictions. Our evaluation of the pilot implementation was limited to a survey of the assessment panel at the time of assessment. Future evaluation of the tool and assessment process should include views of other stakeholders, particularly senior decision-makers within local government and consideration of its value over time, with a particular focus on understanding the contribution of the tool and assessment process to policy processes, and the experience of local government staff in utilising the tool. We also acknowledge that, in this study, our conceptualisation of environmentally sustainable foods and beverages was done at a high-level, without a product-level classification system. Future work can extend the classification of foods and beverages to further guide local governments in this area, particularly for developing detailed guidance on food provision and point-of-purchase-related information.

## Conclusions

The Local Food-EPI+ tool and assessment process is a promising mechanism to guide local governments as they develop and implement policies that aim to create healthy, equitable and environmentally sustainable food systems. The tool can serve an important role in accountability and as an approach to improve the characteristics of food systems that can aid reductions of all forms of malnutrition, drive improved population diets, environmental outcomes and social equity. Further work is needed to determine the feasibility of implementing the tool on a broader scale, in varied local government contexts. Further assessment will identify best practice, monitor policy progress, build capacity and collaboration and strengthen efforts to increase accountability for change.

## Supporting information

Ruffini et al. supplementary material 1 Ruffini et al. supplementary material

Ruffini et al. supplementary material 2 Ruffini et al. supplementary material

## Data Availability

The data presented in this study are available on request from the corresponding author. The data are not publicly available due to agreement with participants not to publicly disclose identifiable data.

## Supplementary material

For supplementary material accompanying this paper visit https://doi.org/10.1017/S136898002400140X
