# Supplementary material for: Development of the Local Food Systems Policy Index (Local Food-EPI+) tool and assessment process to benchmark the implementation of local government policies for creating healthy, equitable and environmentally sustainable food systems
Source: Public Health Nutr. 2024 Oct 2;27(1):e191. doi: 10.1017/S136898002400140X (PMC11504496; doi:10.1017/S136898002400140X)
Supplement: Ruffini et al. supplementary material 1 — Ruffini et al. supplementary material [file S136898002400140Xsup001.docx]

## Development of the Local Food Systems Policy Index (Local Food-EPI+) tool and assessment process to benchmark the implementation of local government policies for creating healthy, equitable and environmentally sustainable food systems

## Online Supplementary Information

**Appendix 1: Local Food Systems Policy Index (Local Food-EPI+) Logic model**


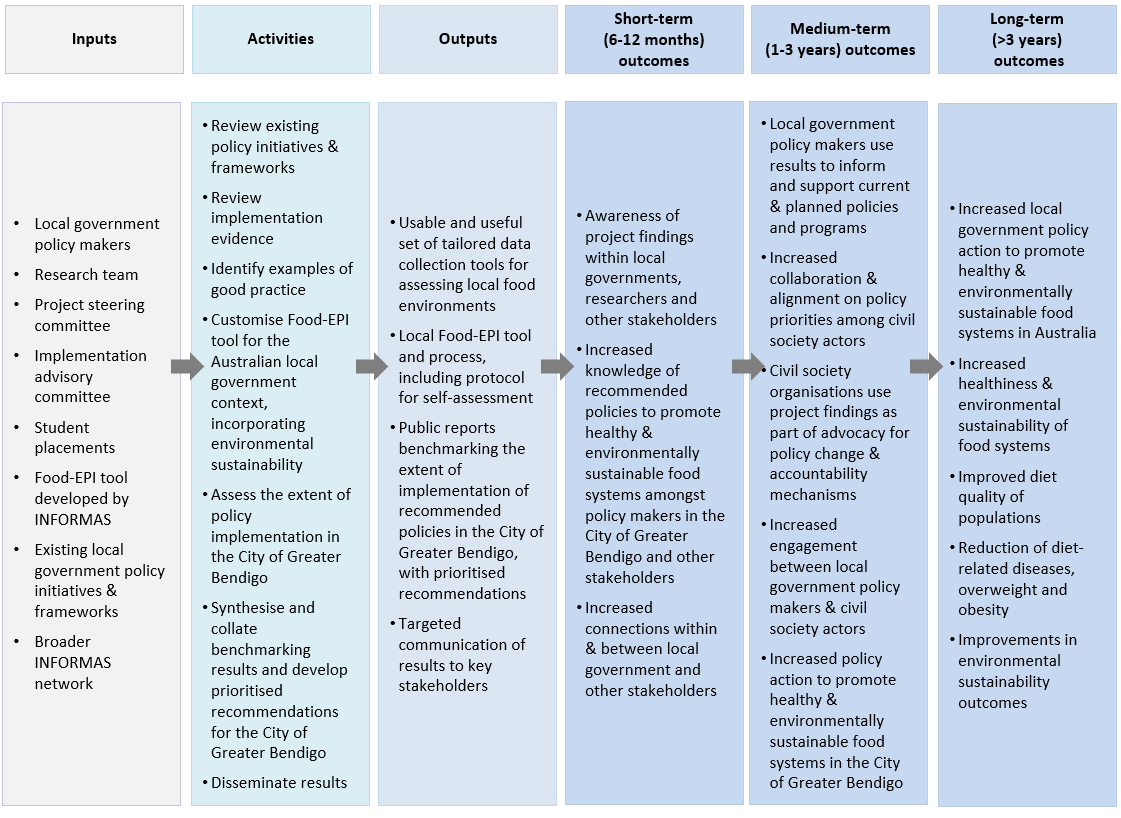


### **Appendix 2: Workshop evaluation questionnaire**

# WORKSHOP EVALUATION

**1. What type of organisation do you primarily work for?**

- Local government
- University or other research institution
- Not-for-profit organisation
- Other _______________________

**2. How would you describe your area of interest (*please tick all that apply*)?**

- Nutrition / health promotion
- Environmental sustainability
- Governance
- Other _______________________

**3. Please rate your level of agreement with the following statements relating to your participation in the workshop today *(please mark one box for each row)*:**

| **As a result of participation in the workshop:** | **Strongly disagree** | **Disagree** | **Neutral** | **Agree** | **Strongly agree** |
| --- | --- | --- | --- | --- | --- |
| a) I have increased knowledge about strategies (at the local government level) to increase the healthiness of food systems and improve population diets |  |  |  |  |  |
| b) I have increased knowledge about strategies (at the local government level)  to increase the environmental sustainability of food systems |  |  |  |  |  |
| c) I have increased knowledge about potential synergies between environmental sustainability and nutrition/health |  |  |  |  |  |
| d) I have increased knowledge of current best practice/what governments are doing locally |  |  |  |  |  |
| e) I have increased knowledge of current best practice/what governments are doing internationally |  |  |  |  |  |
| f) I have made new connections or strengthened existing relationships with government and non-government professionals |  |  |  |  |  |

**4. To what extent might the information discussed today be useful in your work? Please comment.**

**5. Overall, how easy/ difficult did you find it to rate the City of Greater Bendigo?**

- Easy
- Fairly easy
- Fairly difficult
- Difficult

**6. Please tell us what you liked about the Local Food-EPI tool and assessment process:**

**7. Please tell us what you disliked about the Local Food-EPI tool and assessment process, and your suggestions for improvement:**

**8. Please tell us if you think the Local Food-EPI tool and assessment process would be useful in your work and if not, why not?**

**We are aiming for the Local Food-EPI tool and assessment process to be applied by local councils broadly across Victoria and beyond.**

**9. Do you think this would be worthwhile and, if so, what suggestions do you have on ways to facilitate uptake?**

**10. Please provide any other information you would like to share:**

**Appendix 3: Local Food Systems Policy Index (Local Food-EPI+) tool**

Domain: Leadership

| **LEAD1** Strategy and implementation plans for creating and maintaining healthy, equitable and environmentally sustainable food systems and improving population nutrition | |
| --- | --- |
| **Policy objective**  There is a high-level focus, detailed strategy and associated implementation plans for creating and maintaining healthy, equitable and environmentally sustainable food systems, improving population nutrition, and preventing diet-related diseases, including prioritisation for reducing inequalities and supporting vulnerable populations | |
| **LEAD1.1** | **Indicator:**  There is strong, visible high-level support (e.g., vision statements, signatories to international agreements) for creating and maintaining healthy, equitable and environmentally sustainable food systems, improving population nutrition, and preventing diet-related diseases |
| **LEAD1.2** | **Indicator:**  There are over-arching goals in place (including specific, measurable and time-bound targets, e.g., reduce childhood obesity by 10% in 2030) for creating and maintaining healthy, equitable and environmentally sustainable food systems, improving population nutrition, and preventing diet-related diseases |
| **LEAD1.3** | **Indicator:**  There is a detailed strategy for creating and maintaining healthy, equitable and environmentally sustainable food systems, improving population nutrition, and preventing diet-related diseases |
| **LEAD1.4** | **Indicator:**  There is a comprehensive implementation plan supporting strategies for improving population nutrition and environmental sustainability that is adequately resourced, with annual performance and process targets |
| **LEAD1.5** | **Indicator:**  Explicit priority is given to reducing diet-related health inequalities and supporting vulnerable populations as part of relevant strategies, policies and implementation plans |

| Domain: Governance and platforms for engagement**GOVER1** Structures and platforms for collaboration, engagement and cohesion | |
| --- | --- |
| **Policy objective**  The council has governance structures and platforms for engagement that promote collaboration and cohesion across departments, levels of government, and the broader community, as part of efforts to create healthy, equitable and environmentally sustainable food systems, improve population nutrition, and prevent diet-related diseases | |
| **GOVER1.1** | **Indicator:**  Collaborate effectively between **council departments** to ensure a consistent and cohesive approach to creating healthy, equitable and environmentally sustainable food systems, improving population nutrition, and preventing diet-related diseases across all council activities (e.g., through interdepartmental working groups) |
| **GOVER1.2** | **Indicator:**  Actively participate in relevant networks (e.g., involving multiple councils, multiple levels of government, academic experts) to share knowledge and experiences related to efforts to create healthy, equitable and environmentally sustainable food systems, improve population nutrition, and prevent diet-related diseases |
| **GOVER1.3** | **Indicator:**  Systems are in place that promote transparent communication and engage the **community, including indigenous peoples**, in all stages of policy and/or strategy related efforts to create healthy, equitable and environmentally sustainable food systems, improve population nutrition, and prevent diet-related diseases, ensuring minority groups are included |
| **GOVER1.4** | **Indicator:**  There are robust procedures to identify and manage conflicts of interest related to policy development and implementation |

| **GOVER2** Use of evidence to inform policy | |
| --- | --- |
| **Policy objective**  The council uses the best-available evidence to inform policy decisions | |
| **GOVER2.1** | **Indicator:**  Policies and procedures are implemented for using evidence in the development and refinement of policies, including a commitment of routine evaluation of programs |

Domain: Funding and resources

| **FUND1** Government workforce to create and maintain healthy, equitable and environmentally sustainable food systems, and improve population nutrition | |
| --- | --- |
| **Policy objective**  The council has sufficient capacity (number of staff and their capabilities) dedicated to creating and maintaining healthy, equitable and environmentally sustainable food systems, improving population nutrition, and preventing diet-related diseases | |
| **FUND1.1** | **Indicator:**  The council has sufficient capacity (number of staff and their capabilities) dedicated to creating and maintaining healthy, equitable and environmentally sustainable food systems, improving population nutrition, and preventing diet-related diseases, including at least one dedicated food systems officer |

| **FUND2** Funding to create and maintain healthy, equitable and environmentally sustainable food systems, and improve population nutrition | |
| --- | --- |
| **Policy objective**  The council dedicates sufficient funding to creating and maintaining healthy, equitable and environmentally sustainable food systems, improving population nutrition, and preventing diet-related diseases | |
| **FUND2.1** | **Indicator:**  The council has sustained (‘core’) funding to support efforts to create and maintain healthy and environmentally sustainable food systems, including funding for relevant community engagement, monitoring and reporting, policy /program development, and strategic planning |
| **FUND2.2** | **Indicator:**  The council has sufficient funding to promote and support community-led initiatives for creating healthy, equitable and environmentally sustainable food systems, improving population nutrition, and preventing diet-related diseases |

Domain: Monitoring and intelligence

* Monitoring does not need to be conducted by the council; data can be drawn from other sources if available

| **MONIT1** Monitoring of local food environments | |
| --- | --- |
| **Policy objective**  The council routinely monitors and reports on the healthiness, equity and environmental sustainability of local food systems | |
| **MONIT1.1** | **Indicator:**  Routinely monitor and report the relative density of healthy food outlets versus unhealthy food outlets, by geographic area (e. g. suburb, activity area)  ALSO SEE ‘SUPERMARKET AND FOOD SOURCES IN THE COMMUNITY’ POLICY AREA |
| **MONIT1.2** | **Indicator:**  Routinely monitor and report the price and affordability of culturally-appropriate baskets of healthy (compared with unhealthy) foods and beverages, by geographic area (e.g., suburb, activity area) |
| **MONIT1.3** | **Indicator:**  Routinely monitor and report the availability of healthy (compared with unhealthy) food and beverages in food outlets operating in council owned/ managed facilities  ALSO SEE ‘FOOD PROVISION’ AND RETAIL IN COUNCIL FACILITIES AND PUBLIC SPACES POLICY AREA |
| **MONIT1.4** | **Indicator:**  Routinely monitor and report the characteristics of food advertising (e.g., healthy versus unhealthy ads) and sponsorship in public settings, including in and around public transport, schools, sport and recreation facilities, and council owned/managed settings, by geographic area (e.g., suburb, activity area)  ALSO SEE ‘FOOD PROMOTION’ POLICY AREA |
| **MONIT1.5** | **Indicator:**  Routinely monitor and report on levels of food waste, by geographic area (e.g., suburb, activity area)  ALSO SEE ‘FOOD WASTE ‘RE-USE, REDISTRIBUTION AND REDUCTION’ POLICY AREA |
| **MONIT1.6** | **Indicator:**  Routinely monitor and report the use of unsustainable food packaging and/or single-use plastics (e.g., monitor waste streams from businesses who use council waste services, and by incorporating monitoring into Environmental Health Officer food premises compliance checks) |

| **MONIT2** Monitoring of population diets, food system environmental sustainability and related health outcomes | |
| --- | --- |
| **Policy objective**  The council routinely assesses, reports and analyses population diets and related health outcomes | |
| **MONIT2.1** | **Indicator:**  Routinely assess, report and analyse population food and nutrient intake, including analysis by geographic area and differing socio-economic and cultural groups by drawing on relevant existing datasets, supplemented by additional primary data collection if necessary |
| **MONIT2.2** | **Indicator:**  Routinely assess, report and analyse population health outcomes related to nutrition, including analysis by geographic area and differing socio-economic and cultural groups by drawing on relevant existing datasets, supplemented by additional primary data collection if necessary |

Domain: Food production and supply chain

| **PRODS1** Sustainable food production and land management | |
| --- | --- |
| **Policy objective**  The council supports a thriving, diverse and resilient agricultural sector where sustainable land management practices ensure natural resources are protected and enhanced (where applicable) | |
| **PRODS1.1** | **Indicator:**  Develop and implement strategies and policies (with associated education resources, tailored for culturally diverse communities) that support a thriving, diverse and resilient agricultural sector where sustainable land management practices (e.g., cooperative farming ventures, agricultural zoning, vegetated buffers) ensure natural resources are protected and enhanced, and sustainable farming is promoted (e.g., rotational grazing, protection of remnant vegetation/ revegetation with indigenous species to encourage biodiversity, appropriate crops, protection of natural waterways, agroecology, and regenerative agriculture) (where applicable) |
| **PRODS1.2** | **Indicator:**  Develop and implement strategies and policies to protect agricultural land (e.g., systematically mapping land that could be used for food growing) **(where applicable)** |
| **PRODS1.3** | **Indicator:**  Provide resources and/or guidelines to ensure producers, processors, food retailers and caterers are trained and supported on ways to improve energy, water and other resource efficiency across the food supply chain, including tailored support for culturally diverse communities |

| **PROD2** Support for urban agriculture | |
| --- | --- |
| **Policy objective**  The council supports urban agriculture (e.g., community gardens, market gardens, public place plantings, and keeping animals e.g., bees, chickens) | |
| **PRODS2.1** | **Indicator:**  Develop and implement policies and / or programs (e.g., community gardens policy, urban agriculture policy, relevant training) that support urban agriculture on council owned or managed land (e.g., community gardens, market gardens, public place plantings and supporting keeping animals, such as chickens and bees) |

Domain: Food promotion

| **PROMO1** Protection of children from exposure to unhealthy promotion activities | |
| --- | --- |
| **Policy objective**  The council contributes to efforts to ensure that children (including adolescents) are not exposed to the promotion of unhealthy foods and beverages and related brands | |
| **PROMO1.1** | **Indicator:**  Develop and implement policies to restrict the exposure of children (including adolescents) to the promotion of unhealthy foods and beverages ^*^ and related brands in council owned / managed settings **(non-retail)** |

^*^ Unhealthy foods are those considered ‘discretionary’ foods based on the Australian Dietary Guidelines. They are typically high in energy, added sugar, sodium and/or harmful fats, and are often ‘ultra-processed’

| **PROMO2** Healthy sport sponsorship | |
| --- | --- |
| **Policy objective**  The council contributes to efforts to ensure that recreation facilities, sports clubs and associations are free from sponsorship by brands associated with unhealthy foods and beverages | |
| **PROMO2.1** | **Indicator:**  Develop and implement policies (e.g., criteria for accessing facilities, eligibility for council grants) to incentivise recreation facilities, sports clubs and associations to eliminate sponsorship from brands related to unhealthy foods and beverages |
| **PROMO2.2** | **Indicator:**  Provide comprehensive guidance and support to recreation facilities, sports clubs and associations to restrict sponsorship from brands related to unhealthy foods and beverages, including tailored support for culturally diverse communities |

Domain: Food provision and retail in council facilities and public spaces

| **PROV1** Healthy and environmentally sustainable food procurement, provision and catering | |
| --- | --- |
| **Policy objective**  The council ensures implementation of clear, consistent policies for healthy and environmentally sustainable food procurement, provision and catering in council owned/ managed facilities and events | |
| **PROV1.1** | **Indicator:**  Develop and adopt targets for the proportion of food and beverage procured by council (across all relevant operations) that is healthy and environmentally sustainable*  * In considering the environmental sustainability of foods and beverages, the focus is on reducing the environmental impact of products and dietary patterns, including through the way food is produced and transported, minimising food waste and food packaging, and preferencing fresh, minimally-processed seasonal food. |
| **PROV 1.2** | **Indicator:**  Develop and implement a strategy to ensure that council food and beverage procurement activities contribute to improvements in the environmental sustainability of food supply chains (including considerations related to, amongst others, emissions, biodiversity, water usage and discharge, energy usage, environmental compliance, animal-based products, climate change adaptation strategies) |
| **PROV 1.3** | **Indicator:**  Develop and implement a clear, consistent healthy and environmentally sustainable food policy for **food outlets operating in council owned/ managed facilities** (e.g., vendors in sport and recreational centres), including criteria related to availability, accessibility, affordability and promotion |
| **PROV1.4** | **Indicator:**  Develop and implement a clear, consistent healthy and environmentally sustainable food policy related to **foods and beverages provided/sold at council owned/ managed community events**, including criteria related to availability, accessibility, affordability and promotion |
| **PROV1.5** | **Indicator:**  Develop and implement a clear, consistent healthy and environmentally sustainable food policy related for **internal food and beverage provision** (e.g., staff meetings, vending machines in council offices) that applies in all council owned/ managed facilities, including criteria related to availability, accessibility, affordability and promotion |
| **PROV1.6** | **Indicator:**  Provide comprehensive guidance and support for healthy and environmentally sustainable food and beverage provision and catering in council owned/ managed facilities and events, including tailored support for culturally diverse communities |

| **PROV2** Access to free drinking water | |
| --- | --- |
| **Policy objective**  The council ensures accessibility of free drinking water in public spaces and venues | |
| **PROV2.1** | **Indicator:**  Develop and implement policies and/or programs that ensure accessibility of free drinking water in public spaces and venues (e.g., provide free water stations in outdoor publicly accessible places such as parks, include water fountains within new building developments) |

Domain: Supermarkets and food sources in the community

| **RETAIL1** Availability and accessibility of healthy and environmentally sustainable food and beverage retail outlets | |
| --- | --- |
| **Policy objective**  The council encourages the availability and accessibility of healthy and environmentally sustainable food and beverage retail outlets | |
| **RETAIL1.1** | **Indicator:**  Develop and implement policies and/or programs that encourage the availability (particularly in low-income neighbourhoods) and accessibility (e.g., opening hours) of healthy and environmentally sustainable food and beverage retail outlets (e.g., those selling mainly fresh fruit and vegetables) |
| **RETAIL1.2** | **Indicator:**  Develop and implement policies and/or mechanisms to support local food producers to link to local hospitality businesses, farmers’ markets and consumers, maximising fresh food distribution across multiple settings (e.g., farm gate stalls, produce box schemes and farm to table dining) |
| **RETAIL1.3** | **Indicator:**  Provide policy and planning support to ensure that there are public transport/active transport options linking healthy food retail outlets and residential areas |
| **RETAIL1.4** | **Indicator:**  Provide policy and planning support to ensure that new residential developments are built close to food retail outlets selling healthy and environmentally sustainable food (where applicable) |
| **RETAIL1.5** | **Indicator:**  Advocate for changes to the State Planning Policy framework that would allow health to be a consideration as part of planning decisions  ALSO SEE ‘MONITORING AND INTELLIGENCE’ POLICY AREA |

| **RETAIL2** Support for supermarkets and other grocery outlets to improve the healthiness of their in-store environments | |
| --- | --- |
| **Policy objective**  The council works with supermarkets and other grocery outlets to create healthy, equitable and environmentally sustainable in-store retail food environments | |
| **RETAIL2.1** | **Indicator:**  Implement programs to work with supermarkets and other grocery outlets to incentivise their customers to purchase and consume healthy food (e.g., fruit and vegetables) and disincentivise unhealthy options, through strategies such as pricing, promotion and product placement |

| **RETAIL3** Support for food retail outlets (non-grocery) to improve the healthiness of their in-store environments | |
| --- | --- |
| **Policy objective**  The council works with food retail outlets (non-grocery) to create healthy, equitable and environmentally sustainable in-store retail food environments | |
| **RETAIL3.1** | **Indicator:**  Implement programs to work with food retail outlets (non-grocery) to improve the in-store availability, affordability and promotion of healthy foods and reduce the availability and promotion of unhealthy foods |

| **RETAIL4** Support for provision of point-of-purchase nutrition and environmental sustainability information | |
| --- | --- |
| **Policy objective**  The council supports provision of easy-to-understand nutrition and environmental sustainability-related information at point of purchase | |
| **RETAIL4.1** | **Indicator:**  Develop and implement programs that support provision of simple, easy-to-understand nutrition-related information at point of purchase, including consideration for culturally diverse communities |
| **RETAIL4.2** | **Indicator:**  Develop and implement programs that support provision of simple, easy-to-understand environmental sustainability-related information at point of purchase, including consideration for culturally diverse communities |

| **RETAIL5** Reduction/ reuse of unsustainable packaging materials | |
| --- | --- |
| **Policy objective**  The council supports reductions in unsustainable packaging, including promoting minimal use of packaging and use of recyclable or bio-degradable packaging materials | |
| **RETAIL5.1** | **Indicator:**  Develop and implement effective policies to incentivise the use of environmentally sustainable packaging of food products (e.g., waxing, plant-based wrapping, biodegradable plastics) and/or promote minimal use of packaging materials |
| **RETAIL5.2** | **Indicator:**  Provide comprehensive guidance and support to food retailers to encourage the use of ‘Bring Your Own’ (BYO) or returnable packaging, including tailored support for culturally diverse communities |

Domain: Food waste re-use, redistribution and reduction

| **REDUC1** Food waste re-use, redistribution and reduction | |
| --- | --- |
| **Policy objective**  The council works with industry and the community to implement food waste prevention and reduction schemes and composting initiatives. | |
| **REDUC1.1** | **Indicator:**  Develop and implement strategies and/or policies for council internal operations to prevent and minimise food waste going to landfill (e.g., waste assets infrastructure such as food organics and garden organics bins at council-owned facilities and offices, energy recovery and animal feed |
| **REDUC1.2** | **Indicator:**  Develop and implement strategies and/or policies to support coordinated redistribution and/or donation of healthy food to prevent commercial food waste (e.g., through coordination of community groups or support for smartphone applications) |
| **REDUC1.3** | **Indicator:**  Develop / participate in initiatives that educate and support the community (including producers, processors, retailers, caterers, and the wider community) to prevent and minimise food waste (e.g., supporting distributors to optimise supply chains and inventory systems to avoid generating unnecessary surplus, menu planning, utilising seconds fruit to create new food products, rebates for purchasing home compost/worm farms, and food sharing apps), including tailored support for culturally diverse communities |

Domain: Support for Communities

| **COMM1** Coordinated support for community initiatives to promote healthy, equitable and environmentally sustainable food systems and diets | |
| --- | --- |
| **Policy objective**  The council provides coordinated support for community initiatives to create and maintain healthy, equitable and environmentally sustainable food systems | |
| **COMM1.1** | **Indicator:**  The council provides coordinated support for community initiatives to create and maintain healthy, equitable and environmentally sustainable food systems, including an up-to-date inventory of relevant initiatives, platforms for knowledge sharing and collaboration *, and relevant community grant schemes  * Linking with existing initiatives / platforms where possible |

| **COMM2** Food relief services | |
| --- | --- |
| **Policy objective**  The council provides food-related subsidies, support programs, meal services and nutrition-related guidelines to organisations that provide food relief services to people experiencing food shortages | |
| **COMM2.1** | **Indicator:**  Develop and implement nutritious and affordable social meal provision programs (e.g., meals on wheels, lunch clubs and holiday feeding programs) for vulnerable people who might otherwise go hungry or be at risk of malnutrition |
| **COMM2.2** | **Indicator:**  Provide nutrition-related training, support and/or advice to organisations that provide food relief services to people experiencing food shortages and/or vulnerable populations, drawing on relevant state policies and/ or guidelines where possible |
| **COMM2.3** | **Indicator:**  Ensure that any financial support and/or subsidies (e.g., coordination of food relief network, promotion and funding from community grants) provided to food relief organisations includes considerations of culturally appropriate healthy and environmentally sustainable food |

| **COMM3** Breastfeeding and infant feeding policies, programs and facilities | |
| --- | --- |
| **Policy objective**  The council protects, promotes and supports optimal infant feeding practices including breastfeeding and establishment of healthy eating habits | |
| **COMM3.1** | **Indicator:**  Develop and implement policies and/or programs to ensure a supportive environment is provided for people who breastfeed and/ or express milk (e.g., provision of breastfeeding spaces in council buildings, public space furniture that is suitable/ comfortable for breastfeeding, work with local businesses and organisations to support infant feeding and breastfeeding within workplaces and throughout the community) |
| **COMM3.2** | **Indicator:**  Develop * and implement focused education (e.g., provided by Maternal and Child Health nurses where they are employed by council) to support new parents achieve optimal infant feeding practices including breastfeeding and establishment of healthy eating habits with tailored support for culturally diverse communities  * Drawing on existing initiatives / campaigns (developed by organisations/ associations that have the requisite expertise and evidence base) where possible |
| **COMM3.3** | **Indicator:**  Develop * and implement public education (not wholly focused on mothers) to promote, support and protect breastfeeding and the rights of women to breastfeed infants in public, including tailored support for culturally diverse communities  * Drawing on existing initiatives/ campaigns (developed by organisations/ associations that have the requisite expertise and evidence base) where possible |

| **COMM4** Provision of community facilities for cooking and food preparation | |
| --- | --- |
| **Policy objective**  The council provides community facilities for cooking and food preparation | |
| **COMM4.1** | **Indicator:**  Develop and implement policies and/ or programs that provide, maintain and increase the use of communal infrastructure related to food preparation (e.g., picnic areas, public place barbecues, community hall and sport club kitchens) |
| **COMM4.2** | **Indicator:**  Develop and implement policies (e.g., community buildings policy) to ensure council-owned / managed properties provide adequate food storage and preparation areas for healthy cooking (e.g., no deep fryers installed when a kitchen is built or upgraded) |

| **COMM5** Engagement and training for public and private sector organisations | |
| --- | --- |
| **Policy objective**  The council ensures that there are resources and initiatives to support workplaces to provide healthy food and beverage environments and promote healthy and environmentally sustainable behaviours | |
| **COMM5.1** | **Indicator:**  Support workplaces to provide healthy food and beverage environments and promote healthy and environmentally sustainable behaviours through the provision of resources and the promotion of existing initiatives / campaigns (developed by organisations/ associations that have the requisite expertise and evidence base) (e.g., healthy catering policy templates and guidelines) |

| **COMM6** Social marketing programs and campaigns related to healthy, equitable and environmentally sustainable food systems and improving population nutrition | |
| --- | --- |
| **Policy objective**  The council implements evidence-informed social marketing campaigns related to healthy and environmentally sustainable food systems and improving population diets | |
| **COMM6.1** | **Indicator:**  Implement campaigns and initiatives, developed using best-practice principles for social marketing, to promote healthy and culturally-appropriate environmentally sustainable foods and diets, including tailored support for culturally diverse communities *  * Drawing on existing initiatives/ campaigns (developed by organisations/ associations that have the requisite expertise and evidence base) where possible |
| **COMM6.2** | **Indicator:**  Implement campaigns and initiatives, developed using best-practice principles for social marketing, across a range of media on reducing/discouraging single-use plastics and plastic packaging at the point of sale, pick-up and delivery of food (e.g., public campaigns on reducing or recycling of packaging materials targeting food business and consumers, educational initiatives on choosing ‘nude food’ for school lunchboxes), including tailored support for culturally diverse communities (where applicable) *  * Drawing on existing initiatives/ campaigns (developed by organisations/ associations that have the requisite expertise and evidence base) where possible |

**Appendix 4: Local Food Systems Policy Index (Local Food-EPI+) local government good practice examples identified locally and internationally**

Domain: Leadership

Policy objective: There is a high-level focus, detailed strategy and associated implementation plans for creating and maintaining healthy, equitable and environmentally sustainable food systems, improving population nutrition, and preventing diet-related diseases, including prioritisation for reducing diet-related inequalities and supporting vulnerable populations

| **Indicators** | **Good practice examples** | |
| --- | --- | --- |
|  | **Local** | **International** |
| **LEAD1.1** There is strong, visible high-level support (e.g., vision statements, signatories to international agreements) for creating and maintaining healthy, equitable and environmentally sustainable food systems, improving population nutrition, and preventing diet-related diseases  **LEAD1.2** There are over-arching goals in place (including specific, measurable and time-bound targets, e.g., reduce childhood obesity by 10% in 2030) for creating and maintaining healthy, equitable and environmentally sustainable food systems, improving population nutrition, and preventing diet-related diseases  **LEAD1.3** There is a detailed strategy for creating and maintaining healthy, equitable and environmentally sustainable food systems, improving population nutrition, and preventing diet-related diseases  **LEAD1.4** There is a comprehensive implementation plan supporting strategies for improving population nutrition and environmental sustainability that is adequately resourced, with annual performance and process targets  **LEAD1.5** Explicit priority is given to reducing diet-related health inequalities and supporting vulnerable populations as part of relevant strategies, policies and implementation plans | Mornington, Victoria (2022-2027): Mornington Peninsula Shire has developed a [Draft Food Economy and Agroecology Strategy](https://s3.ap-southeast-2.amazonaws.com/hdp.au.prod.app.mpen-shape.files/8816/3115/9710/Draft_Food_Economy_and_AgroEcology_Strategy.pdf) that includes a five-year action plan to support the local agricultural economy and build business resilience through sustainable farming practices, biodiversity and minimising waste.  Melbourne and Sydney, Australia (2021- current): The City of Melbourne and the City of Sydney are signatories to international agreements such as the [Milan Urban Food Policy Pact](https://www.milanurbanfoodpolicypact.org/?fbclid=IwAR3RwTcQWELK-WkEmknIuZ67jQm3IBeKBm0xNdql_rzivX6lh7VHyILpi7Y#mufpp-map), supporting food system transformations. | Brighton & Hove, UK (2017): The [Spade to Spoon: Digging Deeper](https://bristolgoodfood.org/wp-content/uploads/2012/03/The-Bristol-good-food-charter.pdf) is a food strategy and action plan, building on the success from the 2006 strategy. Evaluation of the 2006 strategy revealed that 90% of planned activities were implemented. Their vision is to be a city where everyone has the opportunity to eat fresh, healthy food from sustainable sources. The strategy describes 8 key outcomes and 9 aims with specific objectives for each. The 8 outcomes relate to diet, food poverty, community, economy, waste, climate change, natural resources and research and planning.  Bristol, UK (2011): A Food Policy Council was formed by local government in Bristol and in 2012 the [‘Good Food Charter’](https://bristolgoodfood.org/wp-content/uploads/2012/03/The-Bristol-good-food-charter.pdf) was produced as a “call to arms for all relevant stakeholders to adopt the principle of Good Food encompassing seven hallmarks; Good Food is not only tasty, healthy, affordable, but must also be produced and distributed in a way that it is good for nature, good for workers, good for animal welfare and good for local businesses”. |

Domain: Governance and platforms for engagement

Policy objective: The council has governance structures and platforms for engagement that promote collaboration and cohesion across departments, levels of government, and the broader community, as part of efforts to create healthy, equitable and environmentally sustainable food systems, improve population nutrition, and prevent diet-related diseases, using the best-available evidence to inform policy decisions

| **Indicators** | **Good practice examples** | |
| --- | --- | --- |
|  | **Local** | **International** |
| **GOVER1.1** Collaborate effectively between **council departments** to ensure a consistent and cohesive approach to creating healthy, equitable and environmentally sustainable food systems, improving population nutrition, and preventing diet-related diseases across all council activities (e.g., through interdepartmental working groups)  **GOVER1.2** Actively participate in relevant networks (e.g., involving multiple councils, multiple levels of government, academic experts) to share knowledge and experiences related to efforts to create healthy, equitable and environmentally sustainable food systems, improve population nutrition, and prevent diet-related diseases  **GOVER1.3** Systems are in place that promote transparent communication and engage the community, including indigenous peoples, in all stages of policy and/or strategy related efforts to create healthy, equitable and environmentally sustainable food systems, improve population nutrition, and prevent diet-related diseases, ensuring minority groups are included  **GOVER1.4** There are robust procedures to identify and manage conflicts of interest related to policy development and implementation | Victoria (current): There are over 15 councils within Victoria who are members of [Sustain: the Australian food network, a Local Government networking forum](https://sustain.org.au/articles/new-membership-benefits-for-lgas/) where support staff, managers and elected representatives work together to help understand their roles in building better food systems, building their knowledge and networks through events and consultancy services.  Cardinia, Victoria (2016-current): Cardinia Shire Council developed its first food strategy [The Cardinia Food Circles Collective Impact Project](https://www.cardinia.vic.gov.au/info/20031/liveability_health_and_wellbeing/645/cardinia_food_circles_project#section-1-about-this-project) to establish a healthy, sustainable and fair food system for its residents. The council undertook extensive consultation and implemented a participatory food policy making process, guided by a steering committee and key partners from local and regional health organisations, education and research institutions, food industry, community members, not-for-profit organisations and local government staff. | Toronto, Canada (2012): The [Toronto Golden Horseshoe Food and Farming Action Plan](https://www.toronto.ca/wp-content/uploads/2017/10/88b2-foodfarming_actionplan.pdf) is an intervention aimed to establish a collaboration to adopt a common plan which helps the food and farming sector remain viable in the face of land use pressures at the urban-rural interface, as well as infrastructure gaps, rising energy costs, and disjointed policy implementation. It includes five objectives: (i) to grow the food and farming cluster; (ii) to link food, farming and health through consumer education; (iii) to foster innovation to enhance competitiveness and sustainability; (iv) to enable the cluster to be competitive and profitable by aligning policy tools; and (v) to cultivate new approaches to supporting food and farming. All 7 LGAs adopted this overarching action plan as their own local policy to streamline development and reporting. Each municipality contributes funding ($30k CDN per annum) to employ the Executive Director and the part-funding of projects.  Toronto, Canada (2016): [Toronto’s Long-Term Waste Management Strategy](https://www.toronto.ca/wp-content/uploads/2017/10/9803-Final-Long-Term-Waste-Management-Strategy.pdf). The Food Waste Strategy is described within this broader waste reduction strategy as implementing the Love Food Hate Waste Canada campaign, at a local level across the City of Toronto. Participation of stakeholders is described as 'critical' to effective implementation of the strategy. "Thousands of participants from across the City have provided feedback, engaged in dialogue and accessed key project information through a variety of consultation and engagement activities". The consultation occurred over 3 phases; 'Building the foundation', 'Developing the Waste Strategy', and 'Documenting and deciding on this Waste Strategy' and included 4 surveys with 3400 responses, 12 public consultation events with 4200 people, 6 project updates, 4 speaker sessions, 19 meetings with the Stakeholder Advisory Group. |
| **GOVER2.1** Policies and procedures are implemented for using evidence in the development and refinement of policies, including a commitment of routine evaluation of programs |  |  |

Domain: Funding and resources

Policy objective: The council has sufficient capacity (number of staff and their capabilities) and dedicates sufficient funding to creating and maintaining healthy, equitable and environmentally sustainable food systems, improving population nutrition, and preventing diet-related diseases

| **Indicators** | **Good practice examples** | |
| --- | --- | --- |
|  | **Local** | **International** |
| **FUND1.1** The council has sufficient capacity (number of staff and their capabilities) dedicated to creating and maintaining healthy, equitable and environmentally sustainable food systems, improving population nutrition, and preventing diet-related diseases, including at least one dedicated food systems officer | Cardinia, Melbourne (2019- current): A Food Circles Collective Impact and Urban Agriculture Facilitator is employed to undertake enhancing the local food system, contributing to improved food security and diet status of individuals and communities within the shire. |  |
| **FUND2.1** The council has sustained (‘core’) funding to support efforts to create and maintain healthy and environmentally sustainable food systems, including funding for relevant community engagement, monitoring and reporting, policy/program development, and strategic planning  **FUND2.2** The council has sufficient funding to promote and support community-led initiatives for creating healthy, equitable and environmentally sustainable food systems, improving population nutrition, and preventing diet-related diseases | Canada Bay, New South Wales (2021-current): City of Canada Bay Council has elected to provide direct services to vulnerable populations, recognising the need for access to affordable and nutritious meals to older people by funding part of the salary of a Community Project Officer. |  |

Domain: Monitoring and intelligence

Policy objective: The council routinely monitors and reports on the healthiness, equity and environmental sustainability of local food systems and routinely assesses, reports and analyses population diets and related health outcomes

| **Indicators** | **Good practice examples** | |
| --- | --- | --- |
|  | **Local** | **International** |
| **MONIT1.1** Routinely monitor * and report the relative density of healthy food outlets versus unhealthy food outlets, by geographic area (e. g. suburb, activity area)  **MONIT1.2** Routinely monitor * and report the price and affordability of culturally-appropriate baskets of healthy (compared with unhealthy) foods and beverages, by geographic area (e.g., suburb, activity area)  **MONIT1.3** Routinely monitor * and report the availability of healthy (compared with unhealthy) food and beverages in food outlets operating in council owned/ managed facilities  **MONIT1.4** Routinely monitor * and report the characteristics of food advertising (e.g., healthy versus unhealthy ads) and sponsorship in public settings, including in and around public transport, schools, sport and recreation facilities, and council owned/managed settings, by geographic area (e.g., suburb, activity area)  **MONIT1.5** Routinely monitor * and report on levels of food waste, by geographic area (e.g., suburb, activity area)  **MONIT1.6** Routinely monitor * and report the use of unsustainable food packaging and/or single-use plastics (e.g., monitor waste streams from businesses who use council waste services, and by incorporating monitoring into Environmental Health Officer food premises compliance checks)  * Monitoring does not need to be conducted by the council; data can be drawn from other sources if available | City of Moreland, Victoria (2018-2022): The [Waste and Litter Strategy Action Plan](https://www.moreland.vic.gov.au/globalassets/key-docs/policy-strategy-plan/waste-and-litter-strategy-2018---2022---version-for-web-pdf.pdf) includes ongoing monitoring, by measuring the volume of food waste to landfill, and reporting to refine the kerbside food waste collection program.  City of Manningham, Victoria (2016-2021): [The City Council Food Security Plan 2016-2021](https://www.manningham.vic.gov.au/sites/default/files/uploads/Manningham%20City%20Council%20Food%20Security%20Plan%202016-2021.pdf) includes actions that provide annual updates to the municipal, maps of registered food businesses and the Victorian Healthy Food Basket surveys as a means to monitor the distribution of food businesses categories and affordability. | Vienna, Austria (2012): [The OkoKauf Wien ('EcoBuy') Green Public Procurement program](https://www.wien.gv.at/english/environment/protection/oekokauf/criteria-catalogues.html#food) includes promotion of organic food production, efforts to shorten the supply chain and promotion of seasonal produce. Each of the activities are presented with clear targets and monitoring data.  New York, USA (2010-current): [FoodWorks: a vision to improve NYCs food system](http://council.nyc.gov/press/wp-content/uploads/sites/56/2016/12/foodworks_fullreport_11_22_10.pdf) is a policy that New York City updated in 2013 to present a 59-point plan for a stronger and more sustainable food system. Fundamental gaps in the basic data to measure food system progress were discovered thus legislation was introduced to better understand and monitor changes. The city established metrics across each phase of the food system to measure where food is procured from, benefits to local processors and progress towards eliminating hunger. |
| **MONIT2.1** Routinely assess, report and analyse population food and nutrient intake, including analysis by geographic area and differing socio-economic and cultural groups by drawing on relevant existing datasets, supplemented by additional primary data collection if necessary  **MONIT2.2** Routinely assess, report and analyse population health outcomes related to nutrition, including analysis by geographic area and differing socio-economic and cultural groups by drawing on relevant existing datasets, supplemented by additional primary data collection if necessary  **MONIT2.3** Routinely monitor * and report on food-related greenhouse gas emissions relevant to the local area (NOT FOR ASSESSMENT)  * Monitoring does not need to be conducted by the council; data can be drawn from other sources if available | Melbourne, Victoria (2017-2026): The City of Melbourne’s [Nature in the City; thriving biodiversity and healthy ecosystems strategy](https://www.melbourne.vic.gov.au/SiteCollectionDocuments/nature-in-the-city-strategy.pdf) adopts an action to connect people with community gardens to deepen understanding of food knowledge and environmental awareness. A monitoring program is implemented to assess people’s connection to nature in the city.  Hobsons Bay, Victoria (2013-2030): The [Community Greenhouse Strategy](file:///C:\Users\orianar\Downloads\Community-Greenhouse-Strategy-2013_2030.pdf) focuses on greenhouse gas emissions reductions by implementing household composting programs, with construction guidance and training offered, and monitoring the food waste tonnage going to landfill. | Toronto, Canada (2010): [The Toronto Food Strategy](https://www.toronto.ca/legdocs/mmis/2018/hl/bgrd/backgroundfile-118079.pdf) includes a monitoring framework where strategies in food and health, food and community, and food and environment categories are monitored and outcomes from each category are reported. |

Domain: Food production and supply chain

Policy objective: The council supports urban agriculture and a thriving, diverse and resilient agricultural sector where sustainable land management practices ensure natural resources are protected and enhanced (where applicable)

| **Indicators** | **Good practice examples** | |
| --- | --- | --- |
|  | **Local** | **International** |
| **PRODS1.1** Develop and implement strategies and policies (with associated education resources, tailored for culturally diverse communities) that  support a thriving, diverse and resilient agricultural sector where sustainable land management practices (e.g., cooperative farming ventures, agricultural zoning, vegetated buffers) ensure natural resources are protected and enhanced, and sustainable farming is promoted (e.g., rotational grazing, protection of remnant vegetation/ revegetation with indigenous species to encourage biodiversity, appropriate crops, protection of natural waterways, agroecology, and regenerative agriculture) **(where applicable)**  **PRODS1.2** Develop and implement strategies and policies to protect agricultural land (e.g., systematically mapping land that could be used for food growing) **(where applicable)**  **PRODS1.3** Provide resources and/or guidelines to ensure producers, processors, food retailers and caterers are trained and supported on ways to improve energy, water and other resource efficiency across the food supply chain, including tailored support for culturally diverse communities | Victoria (2021): North Central Catchment Management Authority, Macedon Ranges Shire, Hepburn Shire and City of Greater Bendigo [Healthy Landscapes](https://www.bendigo.vic.gov.au/Services/Business/Growing-our-agriculture-sector/Practical-regenerative-agricultural-communities-program/Regen-Agri-Webinars): Practical Regenerative Agricultural Communities Program aims to raise awareness in the community about sustainable land management practices that improve soil health, reduce exposure to climate risk, enhance biodiversity and increase on-farm productivity. The program includes a series of webinars, field days, on farm workshops and one-to-one support on topics such as grass and pasture identification, compost research and natural capital accounting and climate change. | Baltimore, US (2019): [The Baltimore Sustainability Plan](https://www.baltimoresustainability.org/wp-content/uploads/2019/02/Sustainability-Plan_01-30-19-compressed-1.pdf) includes agriculture land-use policies that encourage urban farms and local food production by creating better defined and supported pathways to ownership and offering incremental opportunities to guarantee long-term land tenure and/or ownership of agricultural spaces, such as “lease to purchase” and other models. |
| **PRODS2.1** Develop and implement policies and/or programs (e.g., community gardens policy, urban agriculture policy, relevant training) that support urban agriculture on council owned or managed land (e.g., community gardens, market gardens, public place plantings and supporting keeping animals, such as chickens and bees) | City of Yarra, Victoria ( 2014-2023): [The Urban Agriculture Strategy](C://Users/orianar/Downloads/Yarra%20Urban%20Agriculture%20Strategy%2020192023.pdf) outlines seven key objects, including support for schools, neighbourhood houses and non-profit community organisations to start or manage communal food gardens with grants, resources and community connections | Cleveland, US (2007): The municipality established as part of their zoning code an [Urban Garden District](http://growingfoodconnections.org/wp-content/uploads/sites/3/gravity_forms/7-1d999688265744f48ad862bf97cf7ab9/2015/04/document-2.pdf) program to ensure that urban garden areas are appropriately located and protected to meet needs for local food production, community health, community education, garden-related job training, environmental enhancement, preservation of green space, and community enjoyment on sites for which urban gardens represent the highest and best use for the community. |

Domain: Food promotion

Policy objective: The council contributes to efforts to ensure that children (including adolescents) are not exposed to the promotion of unhealthy foods and beverages and related brands and that recreation facilities, sports clubs and associations are free from sponsorship by brands associated with unhealthy foods and beverages

| **Indicators** | **Good practice examples** | |
| --- | --- | --- |
|  | **Local** | **International** |
| **PROMO1.1** Develop and implement policies to restrict the exposure of children (including adolescents) to the promotion of unhealthy foods and beverages ^*^and related brands in council owned/ managed settings **(non-retail)**  *Unhealthy foods are those considered ‘discretionary’ foods based on the Australian Dietary Guidelines. They are typically high in energy, added sugar, sodium and/or harmful fats, and are often ‘ultra-processed’  ALSO SEE ‘MONITORING AND INTELLIGENCE’ DOMAIN | Dandenong, Victoria (2019): City of Greater Dandenong [Recreation Reserve Advertising, Promotional and Club Signage Policy](C://Users/orianar/Downloads/Council%20Meeting%20Minutes%20190312%20(A5648262)%20(1).pdf) specifies that signage on council property must not contain direct product advertisements for fast-food products, suppliers and restaurants. The council reserves the right to remove or disallow any advertising it deems inappropriate. | UK, London (2018): The Greater London Authority has banned junk food advertising on London underground, train, tram and bus services, as specified in the [London Food's Strategy report](https://www.london.gov.uk/sites/default/files/final_london_food_strategy.pdf). Only advertisements promoting healthy products are allowed. |
| **PROMO2.1** Develop and implement policies (e.g., criteria for accessing facilities, eligibility for council grants) to incentivise recreation facilities, sports clubs and associations to eliminate sponsorship from brands related to unhealthy foods and beverages  **PROMO2.2** Provide comprehensive guidance and support to recreation facilities, sports clubs and associations to restrict sponsorship from brands related to unhealthy foods and beverages, including tailored support for culturally diverse communities  ALSO SEE ‘MONITORING AND INTELLIGENCE’ DOMAIN | Melbourne, Victoria (2021): City of Port Phillip implemented  the Healthy Choices framework within their [Victorian Public Health and Wellbeing](https://www.portphillip.vic.gov.au/media/uignnz4v/healthy_eating.pdf) Plan which includes the requirement that organisations operating in council owned/ managed facilities do not engage in sponsorship, marketing, branding or advertising of foods and drinks to children and youth that would be inconsistent with the Victorian Government Healthy Choices. | Netherlands, Amsterdam (2018): The Amsterdam Municipality launched an ongoing phased project in 2013 ([Amsterdam Healthy Weight Programme](https://ec.europa.eu/health/sites/default/files/social_determinants/docs/hepp_case-studies_07_en.pdf)) that includes a ban on fast food sponsorship of sporting events held in the Municipality. |

Domain: Food provision and retail in council facilities and public spaces

Policy objective: The council ensures accessibility of free drinking water in public spaces and venues, and implementation of clear, consistent policies for healthy and environmentally sustainable food procurement, provision and catering in council owned/ managed facilities and events

| **Indicators** | **Good practice examples** | |
| --- | --- | --- |
|  | **Local** | **International** |
| **PROV1.1** Develop and adopt targets for the proportion of food and beverage procured by council (across all relevant operations) that is healthy and environmentally sustainable *  *In considering the environmental sustainability of foods and beverages, the focus is on reducing the environmental impact of products and dietary patterns, including through the way food is produced and transported, minimising food waste and food packaging, and preferencing fresh, minimally-processed seasonal food.  **PROV1.2** Develop and implement a strategy to ensure that council food and beverage procurement activities contribute to improvements in the environmental sustainability of food supply chains (including considerations related to, amongst others, emissions, biodiversity, water usage and discharge, energy usage, environmental compliance, animal-based products, climate change adaptation strategies)  **PROV1.3** Develop and implement a clear, consistent healthy and environmentally sustainable food policy **for food outlets operating in council owned/ managed facilities** (e.g., vendors in sport and recreational centres), including  criteria related to availability, accessibility, affordability and promotion  **PROV1.4** Develop and implement a clear, consistent healthy and environmentally sustainable food policy related to **foods and beverages provided/sold at council owned/ managed community events**, including criteria related to availability, accessibility, affordability and promotion  **PROV1.5** Develop and implement a clear, consistent healthy and environmentally sustainable food policy related for **internal food and beverage provision** (e.g., staff meetings, vending machines in council offices) that applies in all council owned/ managed facilities, including criteria related to availability, accessibility, affordability and promotion    **PROV1.6** Provide comprehensive guidance and support for healthy and environmentally sustainable food and beverage provision and catering in council owned/ managed facilities and events, including tailored support for culturally diverse communities  ALSO SEE ‘MONITORING AND INTELLIGENCE’ DOMAIN | Bendigo, Victoria (2018-current): City of Greater Bendigo adopted a [Healthy Food and Catering policy](https://www.bendigo.vic.gov.au/media/5007) (applying to catering, sponsorship, funding, vending machines and gifts) to promote healthy and culturally appropriate food and beverage purchase and consumption choices to employees and the community.  Byron, New South Wales (2022-2026): Byron Shire Council has a [Sustainable Catering Policy](https://www.byron.nsw.gov.au/Council/About-Byron-Shire-Council/Policies/Sustainable-Catering-Policy) that aims to increase the demand for sustainably produced and packaged foods within the Byron Shire and applies to all food procurement and corporate catering arrangements undertaken by Council, including tender or contractual arrangements, up for review in 4 years’ time.  Hawkesbury, New South Wales (2009-current): Hawkesbury Cit Council adopted a [free-range eggs policy](https://www.hawkesbury.nsw.gov.au/for-residents/waste-and-recycling/other-waste-and-recycling-options) to promote the benefits of reducing compost, providing manure and improving mental health for their community | Oxfordshire, England (2022): Oxfordshire County Council has adopted a [plant-based food procurement policy](https://news.oxfordshire.gov.uk/plant-based-food/) where food served to councillors at council catered events is plant-based and with a priority on local sourcing, with plans to explore a broader approach to plant-based and locally sourced food options such as on school menus. This policy is framed by the need to tackle climate change, reduce food waste and support healthy eating.  Barcelona, Spain (2013-2015): Barcelona City Council implemented a [2 year intervention](https://ec.europa.eu/environment/gpp/pdf/news_alert/Issue47_Case_Study99_Barcelona.pdf) to increase the environmental sustainability of food services in 49 kindergartens by introducing sustainability criteria into their catering tenders. Criteria required bidders to provide diversity of foods, limit processed foods, increase plant-based foods, minimise food waste and support sustainable food production practices. Monthly quality controls were conducted with quarterly evaluations by kindergarten staff. |
| **PROV2.1** Develop and implement policies and/or programs that ensure accessibility of free drinking water in public spaces and venues (e.g., provide free water stations in outdoor publicly accessible places such as parks, include water fountains within new building developments) | Bendigo, Victoria (Current): City of Greater Bendigo are mapping their current locations of drinking fountains and installing new drinking fountains to improve accessibility of free drinking water in public spaces and venues.  Sydney, New South Wales (2017- current): City of Sydney has a [Water bubblers](https://www.cityofsydney.nsw.gov.au/lists-maps-inventories/water-bubblers) map available on their website that displays all the drinking fountain locations in parks, playgrounds and public spaces within their municipality | Chicago, USA (2013): [A Recipe for Healthy Places: Addressing the Intersection of Food and Obesity in Chicago](https://www.chicago.gov/content/dam/city/depts/zlup/Sustainable_Development/Publications/Recipe_For_Healthy_Places/Recipe_for_Healthy_Places_Final.pdf) includes a strategy to serve Healthy Food and Beverages (including free water refilling stations to promote tap water consumption) in afterschool and childcare programs. |

Domain: Supermarkets and food sources in the cmmunity

Policy objective: The council works with grocery and non-grocery outlets to create and support the availability and accessibility of healthy, equitable and environmentally sustainable in-store retail food environments, including point of purchase information and support for reductions in unsustainable packaging

| **Indicators** | **Good practice examples** | |
| --- | --- | --- |
|  | **Local** | **International** |
| **RETAIL1.1** Develop and implement policies and/or programs that encourage the availability (particularly in low-income neighbourhoods) and accessibility (e.g., opening hours) of healthy and environmentally sustainable food and beverage retail outlets (e.g., those selling mainly fresh fruit and vegetables)  **RETAIL1.2** Develop and implement policies and/or mechanisms to support local food producers to link to local food procurement activity, hospitality businesses, farmers’ markets and consumers, maximising fresh food distribution across multiple settings (e.g., farm gate stalls, produce box schemes and farm to table dining)  **RETAIL1.3** Provide policy and planning support to ensure that there are public transport/active transport options linking healthy food retail outlets and residential areas  **RETAIL1.4** Provide policy and planning support to ensure that new residential developments are built close to food retail outlets selling healthy and environmentally sustainable food (**where applicable**)  **RETAIL1.5** Advocate for changes to the State Planning Policy framework that would allow health to be a consideration as part of planning decisions  ALSO SEE ‘MONITORING AND INTELLIGENCE’ DOMAIN | Huon Valley, Tasmania (2021): The Huon Valley Council established a [food hub network](https://www.huonvalley.tas.gov.au/grant-awarded-for-supporting-a-huon-valley-thats-healthy-well-and-thriving/) that links local producers and organisations with residents and businesses and provides easier access to healthy food.  Strathfield, New South Wales (2019-current): In response to community concerns about unreliable access to transport connections, parking demand at rail stations and shops and traffic congestion within the Strathfield Council area, Council launched the [Strathfield Connector Bus service](https://www.strathfield.nsw.gov.au/play/strathfield-connector/). The service has two routes servicing north and south and runs seven days a week. This service not only connects residents to local shops, restaurants and businesses but encourages social interaction and access to mobility for those without reasonable access to transport. Since its launch, over 20,000 passengers have used the service.  Cardinia, Victoria (2014-2024): Cardinia Shire Council [Aspirational Energy Transition Plan](https://www.cardinia.vic.gov.au/directory_record/3418/aspirational_energy_transition_plan_2014%E2%80%9324) includes locating high density housing and local food retailers close to rail stations through the planning process as a key ongoing action to address | New York City, USA (2010): [FoodWorks](http://www.ngfn.org/resources/ngfn-database/knowledge/foodworks_fullreport_11_22_10.pdf) policy sets goals to support better choices with a 59 point plan that includes promoting an increase in healthy food retail in existing food deserts, promoting an increase in co-ops offering affordable healthy food and supporting an increase in healthy food availability in convenience stores (e.g. bodegas). |
| **RETAIL2.1** Implement programs to work with supermarkets and other grocery outlets to incentivise their customers to purchase and consume healthy food (e.g., fruit and vegetables) and disincentivise unhealthy options, through strategies such as pricing, promotion and product placement | Bendigo, Victoria (2015-2020): City of Greater Bendigo partnered with the ‘Champions IGA’ retail chain and academic researchers to [trial various in-store changes](https://goodcdn.app/memberhq/vicsport/uploads/Victorian-Healthy-Supermarkets-project-summary-FINAL-1.pdf) (e.g. trolley and basket signage, local area and in-store promotion, and shelf tags highlighting the healthiest packaged foods) to promote a healthier diet. Sales of core foods increased and sales of discretionary foods decreased. | Paris, France (2018): [The Paris Strategy for Sustainable Food](https://www.api-site.paris.fr/paris/public/2018%2F9%2FENG_Abrege_StratAlim.pdf) is made up of 40 actions across 4 themes. The first theme includes access to sustainable food for everyone e.g., mobile grocery stores, prioritise transport infrastructure for businesses offering sustainable food to shorten supply chains and develop sales opportunities, facilitate access to new premises for shops from the social economy and supermarkets to promote local food products. |
| **RETAIL3.1** Implement programs to work with food retail outlets (non-grocery) to improve the in-store availability, affordability and promotion of healthy foods and reduce the availability and promotion of unhealthy foods | Glen Eira, Victoria (2017): The [Glen Eira City Council](https://www.gleneira.vic.gov.au/media/2940/municipal-public-health-and-wellbeing-plan-2017-2021.pdf) worked with Bentleigh-Bayside Community Health to develop a healthy menu criterion that was low in salt, sugar, fat and high in dietary fibre. They awarded 52 premises who met the criteria with a Taste4Health award and 14 premises with a Taste4Health Kids award. | London, UK (2019): [The Pennine Lancashire consortium](https://www.foodactive.org.uk/wp-content/uploads/2019/06/Pennine-Lancs-COTP-Insight-Report-Final.pdf) of local authorities in London developed and tested incentives for takeaways and fast-food businesses to sell and promote healthier food, including free and heavily subsidised advertising, reduced-cost waste management and links to local food growing. |
| **RETAIL4.1** Develop and implement programs that support provision of simple, easy-to-understand nutrition-related information at point of purchase, including consideration for culturally diverse communities  **RETAIL4.2** Develop and implement programs that support provision of simple, easy-to-understand environmental sustainability-related information at point of purchase, including consideration for culturally diverse communities | Melbourne, Victoria (2008 -current): The City of Melbourne implemented a [food labelling traffic light system](https://www.vichealth.vic.gov.au/-/media/ResourceCentre/PublicationsandResources/healthy-eating/Healthy-Choice/City-of-Melbourne-sports-and-recreation-centre-healthy-food-retail-case-study.pdf?la=en&hash=3F631718327A48AD2F31088672B29431497B4293) to classify foods ‘green’ (best choice), ‘amber’ (choose carefully) or ‘red’ (limit intake). The program has expanded from sport and recreation centres and food courts to be fully integrated into events such as Moomba and includes staff and volunteer catering. | Chicago, USA (2013): ‘[A Recipe for Healthy Places](https://www.api-site.paris.fr/paris/public/2018%2F9%2FENG_Abrege_StratAlim.pdf): Addressing the Intersection of Food and Obesity in Chicago’ included a strategy to improve eating habits by engaging grocery chains as partners to promote point of sale messages, healthy food tastings and video messages at cash registers. |
| **RETAIL5.1** Develop and implement effective policies to incentivise the use of environmentally sustainable packaging of food products (e.g., waxing, plant-based wrapping, biodegradable plastics) and/or promote minimal use of packaging materials  **RETAIL5.2** Provide comprehensive guidance and support to food retailers to encourage the use of ‘Bring Your Own’ (BYO) or returnable packaging, including tailored support for culturally diverse communities  ALSO SEE ‘MONITORING AND INTELLIGENCE’ DOMAIN | Tasmania, Hobart (2021-current): A [by-law](https://www.hobartcity.com.au/files/assets/public/rubbish-recycling-and-street-cleaning/l8897-plastic-takeaway-containers-single-use-plastics-2017initiative-single-use-plastic-by-law.pdf) that bans certain single-use plastic food packaging is enforced by The City of Hobart. The by-law is designed to reduce plastic litter and waste going to landfill.  In Western Australia, the state government mandates that retail food businesses are no longer able to supply [plastic food ware](https://plasticsbanwa.com.au/) (applies from July 2022). In Victoria, the state government will prohibit the [sale and use of single-use food ware](https://www.vic.gov.au/single-use-plastics) by February 2023. | San Francisco, USA (2018): [Zero Waste](https://sfenvironment.org/zero-waste-legislation#waste-prevention) is an intervention that encompasses a range of regulatory policies/ordinances, outreach and education. The ordinances include producer responsibility e.g., supporting the state-wide responsibility framework which ensures producers/suppliers are responsible for their waste generation, provides producer responsibility language to be used in city purchasing contracts, prohibiting Styrofoam and polystyrene use and instead requires the use of compostable or recyclable food ware, prohibits use of single-use food service ware and requires reusable cups at events on city property. |

Domain: Food waste re-use, redistribution and reduction

Policy objective: The council works with industry and the community to implement food waste prevention and reduction schemes and composting initiatives

| **Indicators** | **Good practice examples** | |
| --- | --- | --- |
|  | **Local** | **International** |
| **REDUC1.1** Develop and implement strategies and/or policies for **council internal operations** to prevent and minimise food waste going to landfill (e.g., waste assets infrastructure such as food organics and garden organics bins at council-owned facilities and offices, energy recovery and animal feed)  **REDUC1.2** Develop and implement strategies and/or policies to support co-ordinated redistribution and/or donation of healthy food to prevent commercial food waste (e.g., through co-ordination of community groups or support for smartphone applications)  **REDUC1.3** Develop / participate in initiatives that educate and support the community (including producers, processors, retailers, caterers, and the wider community) to prevent and minimise food waste (e.g., supporting distributors to optimise supply chains and inventory systems to avoid generating unnecessary surplus, menu planning, utilising seconds fruit to create new food products, rebates for purchasing home compost/worm farms, and food sharing apps), including tailored support for culturally diverse communities | Albury, News South Wales (2016- current): [Albury Wodonga Regional Foodshare](https://foodshare.org.au/) collects and redistributes food that would otherwise go to waste, recovering 902,000kg of food in from 44 donors (manufacturers, distributors and local producers).  Melbourne, Victoria (2021): The City of Melbourne [community garden policy](https://www.melbourne.vic.gov.au/residents/home-neighbourhood/gardens-and-green-spaces/Pages/community-gardens-compost-hubs.aspx) includes a community composting scheme which provides a variety of compost hubs across the city where residents can turn food waste into nutrients for soil.  Sydney (2022): Rebates are provided for residents when purchasing compost bins and/or worm farms at Mosman, Randwick, Wingecarribee, Ku-ring-gai Councils. | Amsterdam, The Netherlands (2021): Amsterdam's regional council lead awareness-raising campaigns that include platforms to foster collaboration between stakeholders in the food chain, such as [Too Good to Go](https://toogoodtogo.org/en); an app used to decrease food waste by connecting food from local shops to customers.  The Netherlands, Amsterdam (2020-2025): Municipality of Amsterdam have adopted initiatives such as Waste Transformers (creating decentralised nutrient and energy hubs where residual waste streams are converted into energy and natural resources are recovered) and Biomeiler (creating heat and biogas by composting) as part of their [Amsterdam Circular Strategy](https://assets.website-files.com/5d26d80e8836af2d12ed1269/5de954d913854755653be926_Building-blocks-Amsterdam-Circular-2019.pdf). |

Domain: Support for Communities

Policy objective: The council provides coordinated support mechanisms, resources and facilities for community initiatives to create and maintain healthy, equitable and environmentally sustainable food systems and improve population diets

| **Indicators** | **Good practice examples** | |
| --- | --- | --- |
|  | **Local** | **International** |
| **COMM1.1** The council provides coordinated support for community initiatives to create and maintain healthy, equitable and environmentally sustainable food systems, including an up-to-date inventory of relevant initiatives, platforms for knowledge sharing and collaboration *, and relevant community grant schemes  * Linking with existing initiatives / platforms where possible | Moreland, Melbourne (2017-2020): The [Moreland Food System Strategy](https://www.moreland.vic.gov.au/globalassets/key-docs/policy-strategy-plan/food-systems-strategy--20172020.pdf) included a seeding grant and planning approval to kick start a community food production initiative on a vacant block of council’s land. |  |
| **COMM2.1** Develop and implement nutritious and affordable social meal provision programs (e.g., meals on wheels, lunch clubs and holiday feeding programs) for vulnerable people who might otherwise go hungry or be at risk of malnutrition  **COMM2.2** Provide nutrition-related training, support and/or advice to organisations that provide food relief services to people experiencing food shortages and/or vulnerable populations, drawing on relevant state policies and/ or guidelines where possible  **COMM2.3** Ensure that any financial support and/or subsidies (e.g., coordination of food relief network, promotion and funding from community grants)  provided to food relief organisations includes considerations of culturally appropriate healthy and environmentally sustainable food | South Australia (2021): [The Nutrition Guidelines for the Food Relief Sector in South Australia](https://www.sahealth.sa.gov.au/wps/wcm/connect/public+content/sa+health+internet/about+us/about+sa+health/health+in+all+policies/public+health+partner+authorities/department+of+human+services+sa+dhssa/south+australian+food+relief+charter+and+nutrition+guidelines) were developed to improve the availability of a nutritious food supply to food relief recipients. They are designed to assist food relief providers move towards a healthy food supply and provide examples. | Toronto, Canada (2012-2014): The [Mobile Good Food Market Program](https://www.nycfoodpolicy.org/mobile-good-food-market-program-toronto-urban-food-policy-snapshot/) utilised old unused transit buses converted into mobile grocery stores that sell fresh and high-quality produce at affordable prices to underserved, low-income communities. The program was part of the Toronto Food Policy and reached over 14,200 residents within its first 20 weeks. |
| **COMM3.1** Develop and implement policies and/or programs to ensure a supportive environment is provided for people who breastfeed and/or express milk (e.g., provision of breastfeeding spaces in council buildings, public space furniture that is suitable/comfortable for breastfeeding, work with local businesses and organisations to support infant feeding and breastfeeding within workplaces and throughout the community)  **COMM3.2** Develop * and implement focused education (e.g., provided by Maternal and Child Health nurses where they are employed by council) to support new parents achieve optimal infant feeding practices including breastfeeding and establishment of healthy eating habits with tailored support for culturally diverse communities  **COMM3.3** Develop * and implement public education to promote, support and protect breastfeeding and the right to breastfeed infants in public, including tailored support for culturally diverse communities  * Drawing on existing initiatives / campaigns (developed by organisations/ associations that have the requisite expertise and evidence base) where possible | Fairfield, Sydney (2018-current): [The Fairfield City Health Framework](https://www.fairfieldcity.nsw.gov.au/Community/Healthy-Fairfield?BestBetMatch=breastfeeding\|3cf0ade6-4fea-4c1f-ab80-e0768e8ec32b\|da470fad-4412-40b8-8297-f1368dde619b\|en-AU#section-8) includes support for breastfeeding in public areas, restaurants, new retail and business developments and workplaces. The Fairfield City Council advocated for breastfeeding areas in public spaces by promotion of “Breastfeeding Welcome Here” stickers across Council’s facilities and business.  Whittlesea, Victoria (2020-current): The City of Whittlesea delivers the [INFANT Program](https://www.infantprogram.org/about/#blurb-tabs) to parents with children 3-12 months old, aiming to promote healthy eating behaviours through the key developmental stages in their children, offering support and practical advice. In 2021, the Victorian Department of Health and Human Services provided funding to broaden application to over 30 Victorian councils through the existing Maternal and Child Health Nurse. | London, UK (2018-current): The [London Childhood Obesity Taskforce](https://www.london.gov.uk/what-we-do/health/londons-child-obesity-taskforce#acc-i-56597) (32 local London districts that make up Greater London) includes  peer-to-peer support networks and trial incentives with academic partners to help mothers feel more supported to breastfeed for longer, and in more places, as well as collects  and analyses robust breastfeeding data at ten days and six-to-eight weeks, to explore how mothers can be more supported to breastfeed for longer. |
| **COMM4.1** Develop and implement policies and/ or programs that provide, maintain and increase the use of communal infrastructure related to food preparation (e.g., picnic areas, public place barbecues, community hall and sport club kitchens)  **COMM4.2** Develop and implement policies (e.g., community buildings policy) to ensure council-owned / managed properties provide adequate food storage and preparation areas for healthy cooking (e.g., no deep fryers installed when a kitchen is built or upgraded) | Albury, NSW (2018-current): Albury City council procured [community wood fired ovens](https://www.alburycity.nsw.gov.au/leisure/parks-and-public-spaces/wood-fired-oven) for free community use every Sunday, with a coordinator on hand to help. Albury’s culturally diverse communities gathered to celebrate and showcase their cultural food at events such as Harmony Day and the Hindu celebrations of Holi and Navarati. | Vancouver, Canada (2011): The Metro Vancouver's [Regional Food System Strategy](http://growingfoodconnections.org/wp-content/uploads/sites/3/2018/03/RegionalFoodSystemStrategy.pdf) evaluates opportunities for using facilities in community centres to establish community kitchen facilities to support preparation of healthy meals. |
| **COMM5.1** Support workplaces to provide healthy food and beverage environments and promote healthy and environmentally sustainable behaviours through the provision of resources and the promotion of existing initiatives / campaigns (developed by organisations/ associations that have the requisite expertise and evidence base) (e.g., healthy catering policy templates and guidelines) | Bendigo, Victoria (2022): Greater Bendigo implemented a [Healthy Catering Guide](https://www.bendigo.vic.gov.au/sites/default/files/2021-11/Greater%20Bendigo%27s%20Healthy%20Catering%20Guide_A4.pdf) for workplaces across the Greater Bendigo municipality, as part of a broader food strategy. | Baltimore, USA, (2019): The Baltimore City Council partnered with private and public sectors to support the [Baltimore Food Hub](http://baltimorearchitecture.org/wordpress/wp-content/Food-Hub-Leasing-Brochure_March-2019_pages.pdf), a campus of job-training facilities, communal incubator space, land dedicated to urban farming, services and programs focused on enhancing Baltimore’s local food economy. |
| **COMM6.1** Implement programs and campaigns, developed using best-practice principles for social marketing, to promote healthy and culturally-appropriate environmentally sustainable foods and diets, including tailored support for culturally diverse communities *  **COMM6.2** Implement programs and campaigns, developed using best-practice principles for social marketing, across a range of media on reducing/discouraging single-use plastics and plastic packaging at the point of sale, pick-up and delivery of food (e.g., public campaigns on reducing or recycling of packaging materials targeting food business and consumers, educational initiatives on choosing ‘nude food’ for school lunchboxes), including tailored support for culturally diverse communities **(where applicable) ***  * Drawing on existing programs / campaigns (developed by organisations/ associations that have the requisite expertise and evidence base) where possible | Knox, Victoria, (2013): Healthy Together Knox, in partnership with Knox City Council Health Services and local retailers encouraged the community to seek out healthier options via their “[Vegspiration](http://www.thereputationgroup.com.au/vegspiration)” campaign and “Knox Friends for Health” Facebook page. Cooking demonstrations and recipe cards were distributed through community avenues and traction to the Facebook page exceeded set targets.  New South Wales (2017-current): Northern Beaches Council’s [Swap This For That](https://www.northernbeaches.nsw.gov.au/services/rubbish-and-recycling/single-use-plastic-reduction/swap-this-for-that) campaign has a strong focus on reducing the use and littering of single-use plastics, including food packaging. The campaign encourages the community to refuse single use plastics bags, bottles, straws, cutlery, plates and coffee cups and use sustainable alternatives. It builds capacity for businesses and community to move away from single use plastics by promoting alternatives and providing resources and education.  Banyule, Victoria (2019-2023): The Banyule City Council’s [Towards Zero waste Plan](https://www.banyule.vic.gov.au/About-us/Policies-plans-strategies/Council-plans-and-strategies/Zero-waste-plan#:~:text=The%2010%2Dyear%20plan%20is,plan%20has%20four%20strategic%20directions.) aims to support and strengthen a community culture to strive for zero waste, promoting where to recycle items not accepted in the household recycling collection through multiple streams including the council’s Facebook page, website, local paper, letters to the households, information cards handed out at community events and utilising a community group using their networks. The council received 842 responses with 59% from letters to the households. | California, USA (2015): [Drink Tahoe Tap](https://www.sei.org/wp-content/uploads/2021/02/210216-caldwell-sle-plastics-report-with-annex-210211.pdf) campaign run by a coalition of 40 organisations focused on keeping Lake Tahoe clean and free of plastic. The campaign focuses on asking people to drink tap water rather than bottled water, refuse single-use plastic products and packaging and to choose reusable packaging and food ware. The campaign is promoted through social media and website channels and in-store signage around Tahoe, using engaging messaging using humour. |

**Appendix 5: Excerpts from the Local Food Systems Policy Index (Local Food-EPI+) tailored report for the City of Greater Bendigo. The full report is available for the authors on request.**


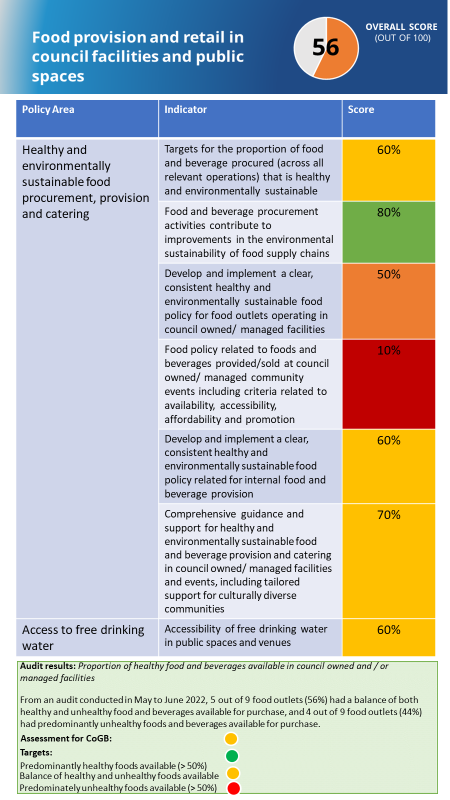

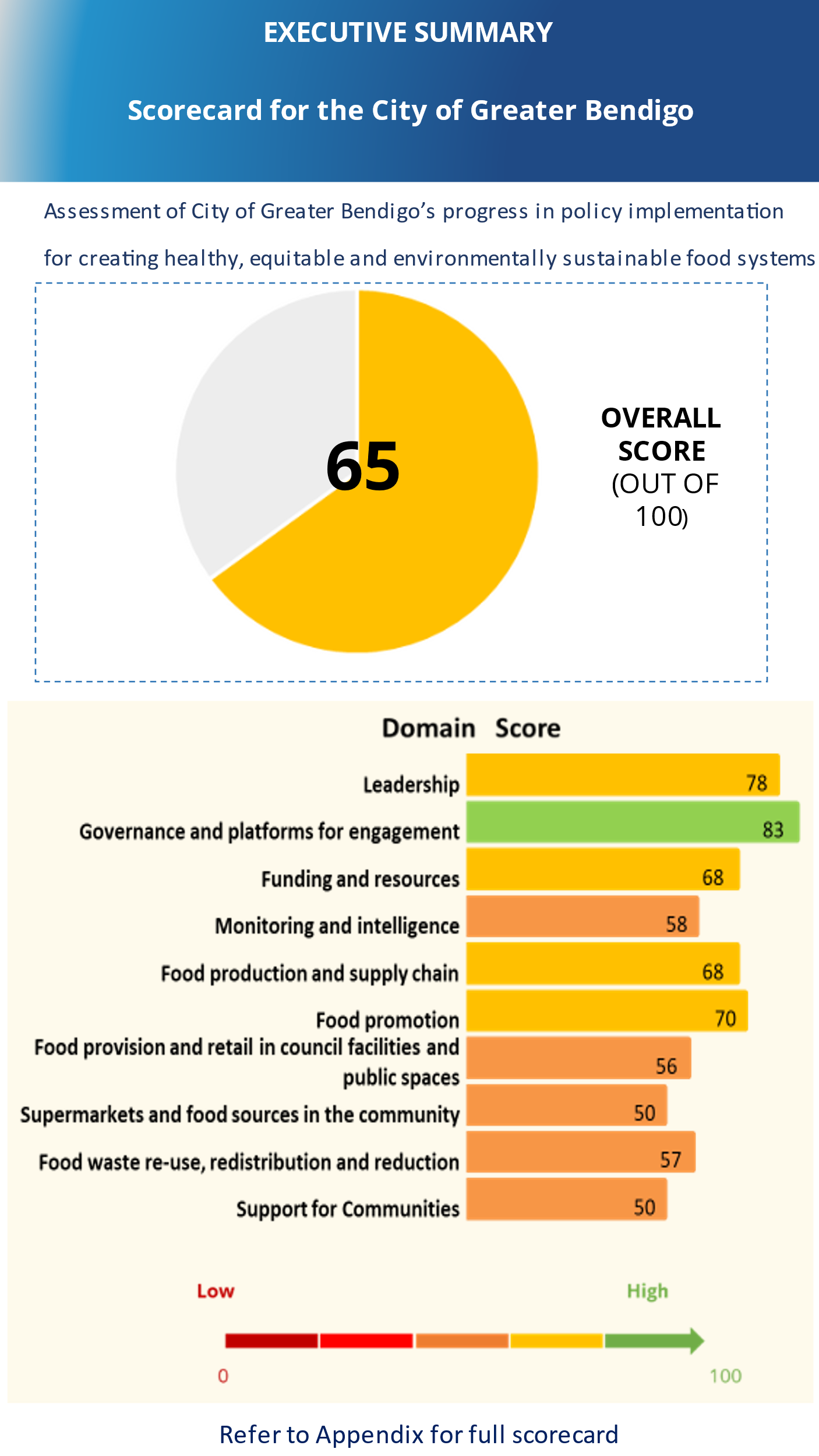


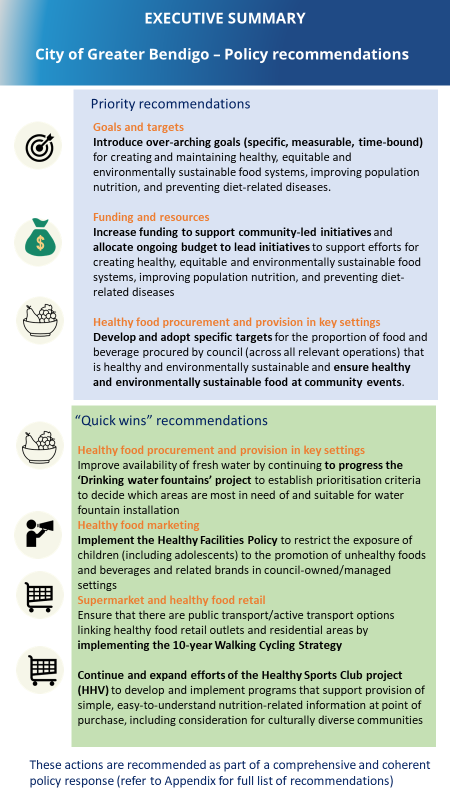

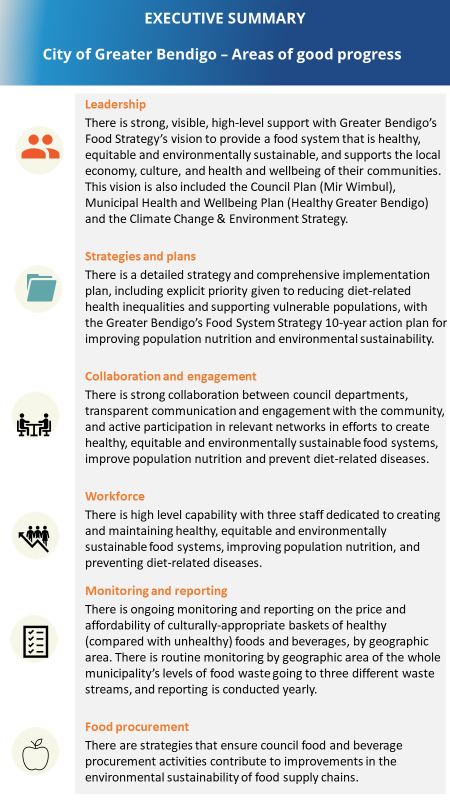


**Appendix 6: Workshop evaluation results**

| **As a result of participation in the workshop:** | **Strongly disagree** | **Disagree** | | **Neutral** | | **Agree** | | **Strongly agree** |
| --- | --- | --- | --- | --- | --- | --- | --- | --- |
| I have increased knowledge about strategies (at the local government level) to increase the healthiness of food systems and improve population diets |  |  | |  | | 9 (45%) | | 11 (55%) |
| I have increased knowledge about strategies (at the local government level)  to increase the environmental sustainability of food systems |  |  | | 1 (5%) | | 10 (50%) | | 9 (45%) |
| I have increased knowledge about potential synergies between environmental sustainability and nutrition/health |  |  | | 6 (30%) | | 9 (45%) | | 5 (25%) |
| I have increased knowledge of current best practice/what governments are doing locally |  |  | | 3 (15%) | | 7 (35%) | | 10 50%) |
| I have increased knowledge of current best practice/what governments are doing internationally |  |  | | 8 (40%) | | 9 (45%) | | 3 (15%) |
| I have made new connections or strengthened existing relationships with government and non-government professionals | 1 (5%) | 6 (30%) | | 5 (25%) | | 7 (35%) | | 1 (5%) |
|  | **Difficult** | | **Fairly difficult** | | **Fairly easy** | | **Easy** | |
| Overall, how easy/ difficult did you find it to rate the City of Greater Bendigo? | 1 (5%) | | 2 (10%) | | 14 (70%) | | 3 (15%) | |
